# Alternative splicing of synaptotagmin 7 regulates oligomerization and short-term synaptic plasticity

**DOI:** 10.1101/2025.10.27.684894

**Published:** 2025-10-28

**Authors:** Nikunj Mehta, Devin T. Larson, Raghava Jagadeesh Salaka, Mitch Wozney, Smrithika Subramani, Shweta Mishra, Simi Kaur, Avani Jain, Edwin R. Chapman

**Affiliations:** 1 Department of Neuroscience, University of Wisconsin-Madison, Madison, WI, United States.; 2 Biophysics Graduate Program, University of Wisconsin-Madison, Madison, WI, United States.; 3 Howard Hughes Medical Institute, Madison, WI, United States.; 4 Wisconsin School of Business, University of Wisconsin-Madison, Madison, WI, United States.

**Keywords:** Short-term plasticity, liquid-liquid phase separation, synaptotagmin, paired-pulse facilitation, synaptic depression, MINFLUX super resolution microscopy, active zone, iGluSnFR, alternative splicing, glutamate sensing, aggregation

## Abstract

Synaptic plasticity is crucial for learning and memory. The presynaptic calcium sensor synaptotagmin 7 (syt7) regulates aspects of short-term plasticity (STP), but the underlying mechanisms remain unclear. Here, we show that alternative splicing of the syt7 juxtamembrane linker acts as a molecular switch at both biochemical and functional levels. The α and β variants undergo liquid-liquid phase separation to form condensates, while the γ variant forms aggregates. Using iGluSnFR imaging, we found that when expressed at equal levels, these three isoforms also diverge regarding their abilities to regulate two key aspects of STP: paired-pulse facilitation and synaptic depression. Further, MINFLUX super resolution microscopy demonstrated that syt7 forms clusters in the active zone, well-positioned to directly control synaptic vesicle dynamics. Thus, alternative splicing might fine-tune STP by differentially impacting syt7 oligomerization.

## Introduction

Intricate communication between neurons, via synaptic transmission, forms the foundation of neural circuit function and ultimately underlies cognition and behavior. Many kinds of synapses in the central nervous system are endowed with the ability to rapidly and reversibly change the strength of transmission over timescales ranging from milliseconds to seconds, collectively referred to as short-term plasticity (STP). Synaptic facilitation and depression are two forms of STP, where successive action potentials (AP) lead to a transient increase or decrease in the amplitude of the synaptic response, respectively (Katz and Miledi, 1968; Citri and Malenka, 2008; Jackman and Regehr, 2017). These processes are predominantly governed by presynaptic mechanisms that modulate the efficacy of synaptic vesicle (SV) fusion with the plasma membrane (Chang et al., 2018; Jackman et al., 2016; Neher and Brose, 2018; Turecek et al., 2017).

**Figure 1.**
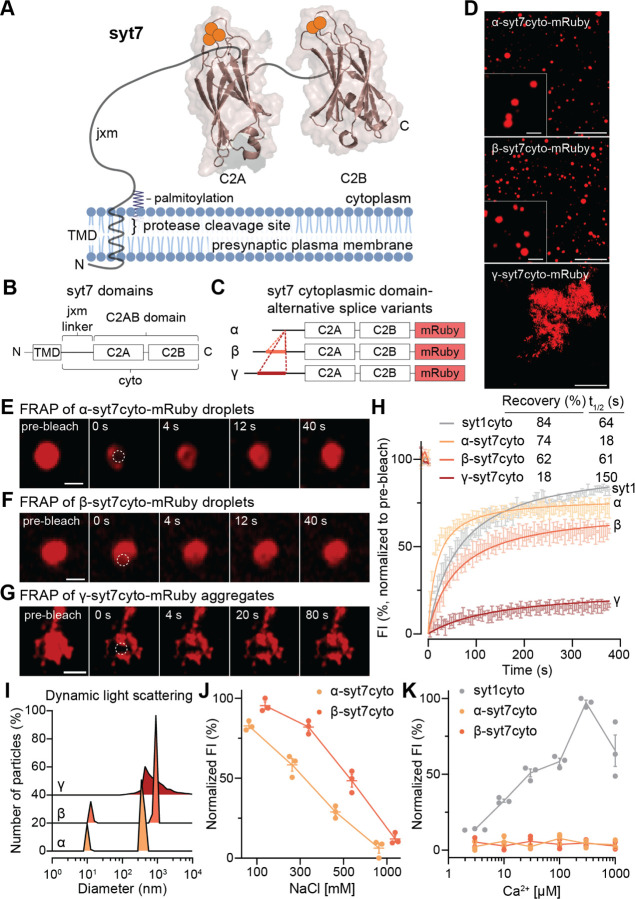
Alternative splicing regulates the oligomerization of synaptotagmin 7. (**A, B**) Illustration of synaptotagmin 7 (syt7), indicating: γ-secretase cleavage within the transmembrane domain (TMD), palmitoylation of the juxtamembrane linker (jxm) that undergoes alternative splicing, and tandem C2 domains, C2A and C2B, that bind Ca^2+^. The membrane was drawn using BioRender, the structures for C2A and C2B (PDB ID: 6ANK) were rendered using UCSF Chimera; Ca^2+^ ions are shown as orange spheres. (**C**) Schematic diagram of the cytoplasmic domain of syt7 (syt7cyto) tagged with a C-terminal mRuby, along with the alternative splice variants: α, β, and γ, which differ only in their jxm linkers. (**D**) α- and β-syt7cyto-mRuby form droplets, whereas γ-syt7cyto-mRuby forms aggregates under the same buffer conditions (protein concentrations: 10 μM α-, β-; 3 μM γ-syt7cyto-mRuby). Zoomed-in α- and β-syt7cyto-mRuby droplets are shown as insets to emphasize the circular shape of droplets. Scale bar, 10 μm. Inset scale bar, 2 μm. (**E-G**) Time series of fluorescence recovery after photobleaching (FRAP) of (E, F) α- and β-syt7cyto-mRuby droplets and (G) γ-syt7cyto-mRuby aggregates. The dotted circle shows the area, with the highest intensity of photobleaching, within the droplet and aggregate. Scale bars: (E, F), 1 μm; (G), 2.5 μm. (**H**) Quantification of fluorescence intensity (FI) from the FRAP time series for the α-, β-, and γ-syt7cyto-mRuby splice variants shown in yellow, orange, and red, respectively. For comparison, FRAP data of jxm-syt1cyto-GFP are replotted, from our previous work (Mehta et al., 2024), in grey. Data were fitted using a hyperbolic function, and the calculated recovery (%) and t_1/2_ (s) values are shown in the inset. (**I**) Number of particles (relative %), as a function of diameter (nm), of syt7cyto splice variant droplets/aggregates determined via dynamic light scattering (DLS). (**J**) Normalized FI of α- and β-syt7cyto-mRuby as a function of [NaCl]. (**K**) Same as (J), but as a function of [Ca^2+^]. For comparison, [Ca^2+^] titration of jxm-syt1cyto-GFP was replotted, from ref. (Mehta et al., 2024), in grey. Three independent trials with 4–10 FOVs per condition were analyzed, data are plotted as mean ± SEM. The buffer used in (D-J) is 25 mM Tris-HCl (pH 7.4), 100 mM NaCl, and 3% PEG 8000; the same buffer, but lacking PEG 8000, was used in panel (K).

SV docking and fusion are regulated by members of the synaptotagmin (syt) family of proteins (Chapman, 2008), characterized by the presence of tandem C2 domains (C2A and C2B) that often serve as Ca^2+^-binding modules (Bhalla et al., 2008; Brose et al., 1992; Wolfes and Dean, 2020), a juxtamembrane (jxm) linker, and a single transmembrane domain (TMD) with a short intralumenal segment (Fig. 1A). The most studied isoform, syt1, is localized to SVs (Matthew et al., 1981) where it plays a role in: SV docking (Chang et al., 2018; Chen et al., 2021; Reist et al., 1998; Wang et al., 2011), clamping release under resting conditions (Bai et al., 2016; Courtney et al., 2019; Littleton et al., 1993), and triggering rapid, synchronous exocytosis in response to increases in [Ca^2+^]_i_ following an AP (Bai et al., 2016; Courtney et al., 2019; Evans et al., 2015; Geppert et al., 1994; Littleton et al., 1993). Another member of this family, syt7, has a similar domain structure but undergoes proteolytic processing to liberate the cytoplasmic domain (Vevea et al., 2021), which is then anchored to the presynaptic plasma membrane via palmitoylation (Flannery et al., 2010).

Syt7 has emerged as a crucial protein essential for two forms of STP: it is required for paired-pulse facilitation (PPF), and it endows synapses with the ability to resist synaptic depression during stimulus trains (Jackman et al., 2016; Turecek et al., 2017; Vevea et al., 2021). Syt7 senses residual [Ca^2+^]_i_ (Jackman and Regehr, 2017), which lingers after an AP, and mediates PPF, owing to its significantly higher Ca^2+^ affinity and slower kinetics, as compared to syt1 (Bhalla et al., 2005; Hui et al., 2005). In addition to playing a key role in STP, syt7 also supports asynchronous release (neurotransmitter release tens to hundreds of milliseconds after an AP) during train stimulus (Bacaj et al., 2013; Vevea et al., 2021; Wen et al., 2010; Wu et al., 2024). More recently, syt7 was proposed to mediate PPF and resistance to synaptic depression by regulating the activity-dependent docking of SVs (Wu et al., 2024). In short, while syt1 provides the synapse with speed and fidelity, syt7 endows synapses with adaptability and endurance.

An emergent area of interest is the potential for syts to form oligomers (Fukuda and Mikoshiba, 2000) and whether oligomerization impacts their function. Indeed, syt1 oligomerizes via its jxm linker to undergo liquid-liquid phase separation (LLPS) (Mehta et al., 2024), and this biochemical property strongly influences both synchronous and spontaneous synaptic transmission (Courtney et al., 2021). Whether syt7 also undergoes LLPS, and how self-association relates to its specific roles in asynchronous release and STP remain largely unexplored. There is some evidence that syt7 can multimerize, but this was proposed to be mediated via its tandem C2-domains (Fukuda et al., 2002a). Interestingly, the jxm linker of syt7 also contains an intrinsically disordered region (IDR) that is potentially conducive to phase separation. Strikingly, this jxm linker is subject to extensive alternative splicing, and three major variants - α, β, and γ - have been described (Fukuda et al., 2002b). However, biochemical and functional differences between these splice variants have yet to be investigated. Finally, while the localization of syt1 to SVs is well established (Brose et al., 1992), the precise localization of syt7 in the plasma membrane of axons is unknown; addressing its spatial organization is needed to understand how syt7 regulates the SV cycle.

The current study aims to investigate the ability of syt7 to oligomerize and to address whether alternative splicing regulates self-association and aspects of STP. By employing iGluSnFR imaging (Marvin et al., 2018), we directly measured glutamate release from cultured neurons and assessed the impact of three syt7 splice variants: α, β, and γ, on STP; in parallel, we confirmed our PPF findings using electrophysiological recordings. Furthermore, we utilize two-color three-dimensional minimal photon flux (MINFLUX) super resolution imaging (Balzarotti et al., 2017; Grabner et al., 2022) to probe the nanoscale organization of syt7 in presynaptic nerve terminals. We report that two splice variants undergo LLPS while one variant forms aggregates. Importantly, these variants also uncoupled the function of syt7 in PPF versus synaptic depression, revealing a functional switch that is subordinate to alternative splicing. Finally, our MINFLUX data reveal that syt7 forms clusters that are positioned to regulate SV dynamics at the active zone.

## Alternative splicing regulates syt7 self-association

Syt7 undergoes alternative splicing in the region that encodes the jxm linker, which connects its calcium-sensing tandem C2-domains to the presynaptic plasma membrane via palmitoylation (Fig. 1A, B; table S1). It is currently thought that there are three major splice variants of syt7, referred to as α, β, and γ (Fig. 1C) (Fukuda et al., 2002b; Sugita et al., 2001). The expression of these isoforms is developmentally and regionally regulated (Sugita et al., 2001), and it has been proposed that they might function differentially during insulin secretion from pancreatic β cells (Gauthier et al., 2008). The discovery that syt1 undergoes LLPS via its jxm linker (Mehta et al., 2024), and that this interaction is crucial for function (Courtney et al., 2021), prompted us to explore whether syt7 also undergoes LLPS. As noted above, the syt7 linker has an IDR and, again, its length and composition are impacted by alternative splicing (table S1). Using mRuby as a C-terminally tagged fluorescent label on the cytosolic (cyto) domain of syt7 (Fig. 1C), we found that the α and β variants formed droplets in aqueous buffer, whereas the γ variant formed amorphous and disordered aggregates under the same conditions (Fig. 1D). To further validate the differential homo-oligomerization of syt7 splice variants, we performed fluorescence recovery after photobleaching (FRAP) on the α- and β-syt7cyto droplets, and γ-syt7cyto aggregates (Fig. 1E–H). FRAP data were fitted to hyperbolic functions to calculate the extent and t_1/2_ of recovery. After bleaching within protein droplets and aggregates (bleaching diameter between 0.8–2 μm), α- and β-syt7cyto droplets rapidly recovered (t_1/2_ = 18, and 61 s, respectively; yellow and orange traces; Fig. 1E,F,H), whereas γ-syt7cyto aggregates recovered slowly (t_1/2_ = 150 s; red trace; Fig. 1G,H). The extent of recovery of γ-syt7cyto aggregates was extremely low (18% recovery), as compared to α- and β-syt7cyto droplets (74 and 62% recovery, respectively; Fig. 1E–H), supporting the idea that α- and β-syt7cyto undergo LLPS to form protein droplets, while γ-syt7cyto again tends to form aggregates. For comparison, FRAP of syt1cyto (80–421 residues), from our previous study (Mehta et al., 2024), is replotted in grey, and exhibited 84% recovery and a t_1/2_ of 64 s (Fig. 1H). To assess the effect of molecular crowding on syt7 splice variants, we characterized droplet formation by each syt7 variant as a function of increasing [PEG 8000] and [protein]. Phase diagrams in fig. S1A–C indicate that β-syt7cyto has a slightly higher propensity to form droplets than α-syt7cyto (at 3 μM [protein] and 3% PEG 8000, and 10 μM [protein] and 1% PEG 8000), whereas γ-syt7cyto forms aggregates under all [protein] and [PEG 8000] conditions. As a control, mRuby and syt7C2AB-mRuby, lacking the jxm linker, did not form droplets under the same conditions (fig. S1D–F), further indicating that the jxm linker drives self-association. Additionally, α-, as well as β-syt7cyto droplets, undergo homotypic fusion, another characteristic property of LLPS droplets (fig. S1G, H).

A classic signature of protein aggregation is high hydrophobicity. Notably, sequence analysis of the jxm linker of the γ-syt7 splice variant showed a higher hydropathicity index (a high index indicates high hydrophobicity) as compared to the jxm linkers of α- and β-syt7 (fig. S2A–C). Size characterization of protein droplets and aggregates by dynamic light scattering (DLS) indicated that the α- and β-syt7cyto droplets had two peaks corresponding to monomeric and higher-order structures, whereas γ-syt7cyto aggregates had a wide range of large diameter structures (Fig. 1I) (note: the DLS instrument cannot distinguish particle sizes >1 μm). As controls, mRuby and syt7C2AB-mRuby showed single monomeric peaks (fig. S3A), establishing that neither the fluorescent tag nor the two C2-domains undergo LLPS under these conditions. To identify the types of interactions within α- and β-syt7cyto droplets, we challenged the droplets with increasing ionic strength and observed that they progressively dissolved (Fig. 1J). These results suggest that electrostatic interactions contribute to their phase separation. In sharp contrast, γ-syt7cyto aggregates were not affected by increasing ionic strength (fig. S3B). We previously found that Ca^2+^ promotes syt1 droplet formation, so we examined the effects of Ca^2+^ on syt7 droplets. As shown in Fig. 1K and fig. S3C, Ca^2+^ did not promote oligomerization of any of the three splice variants. The effect of Ca^2+^ to promote syt1 droplet formation is included for comparison (grey trace; data are replotted from ref. (Mehta et al., 2024)). Collectively, these data show that the β variant has a somewhat greater propensity to undergo LLPS versus α, while the γ variant forms more rigid aggregates.

## Alternative splicing and subcellular localization determine the extent and kinetics of recovery of syt7 after photobleaching

To examine syt7 LLPS in cells, we expressed the cytoplasmic domains of each of the three mRuby-tagged variants in hippocampal neurons and challenged them with 10% 1,6-hexanediol (1,6-HD), an aliphatic alcohol that disrupts weak hydrophobic interactions within droplets to dissolve them. As shown in fig. S4A,B, α- and β-syt7cyto formed droplets in the soma and neurites. Upon addition of 1,6-HD, the condensates dissolved, in agreement with our biochemical observations that α- and β-syt7cyto undergo LLPS. In contrast, 1,6-HD had no effect on clusters formed by γ-syt7cyto, consistent with the idea that this variant forms aggregates in cells (fig. S4C). These observations lead to the question of LLPS in the context of membrane-associated proteins; in this case, we reiterate that after processing, syt7 is attached to the plasma membrane via palmitoylation (Flannery et al., 2010; Vevea et al., 2021). To address two-dimensional (2D) LLPS on a membrane, we attached His-tagged α-syt7cyto-mRuby droplets to the surface of giant unilamellar vesicles (GUVs) using an 18:1 DGS-NTA (Ni) lipid. Fig. S5A,B shows that α-syt7cyto-mRuby assembled into LLPS patches on a GUV, to form a ‘phase-separated surface’.

**Figure 2.**
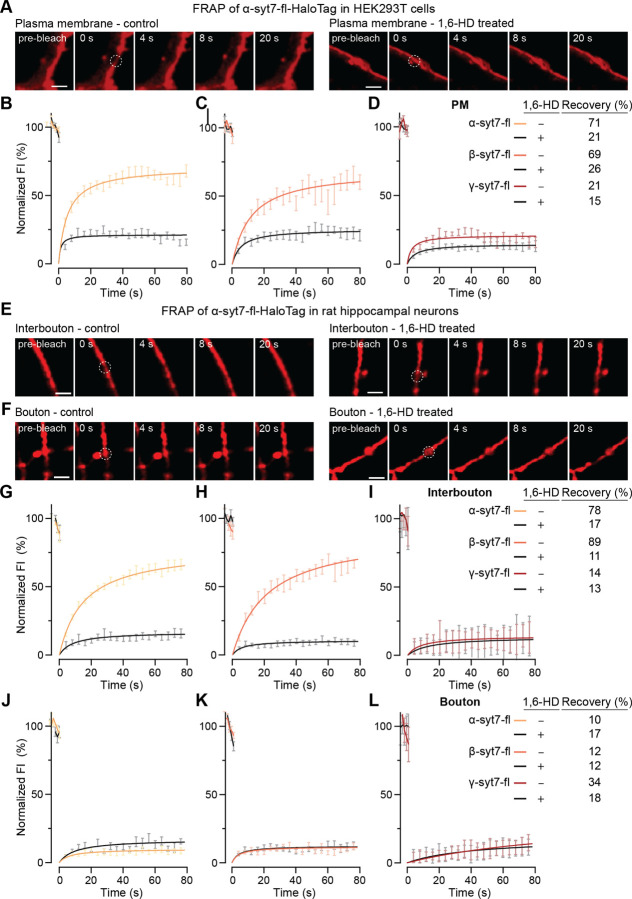
Extent and kinetics of recovery from photobleaching depend on alternate splicing and subcellular localization of syt7 (**A**) Time series of FRAP at the plasma membrane of HEK293T cells expressing α-syt7-full-length (fl)-HaloTag, under control and 10% 1,6-hexanediol (1,6-HD; an aliphatic alcohol that disrupts phase separation of proteins) conditions, respectively. Scale bar, 2 μm. (**B-D**) Quantification of FRAP in HEK293T cells expressing α-, β-, or γ-syt7-fl-HaloTag splice variants in yellow, orange, and red, respectively; the black traces in each panel indicate FRAP experiments with 10% 1,6-HD treatment. Data were fitted using a hyperbolic function to calculate recovery (%) and t_1/2_ (s), as shown in the inset and table S2, respectively. (**E,F**) FRAP time series of α-syt7-fl-HaloTag along axons (interbouton) and within boutons, respectively, in rat hippocampal neurons, under control and 10% 1,6-HD conditions. Scale bar, 2 μm. (**G-I**) and (**J-L**) Same as (B-D), but within interbouton segments versus boutons, respectively. Inset shows % recovery; table S3 indicates calculated t_1/2_ (s) values. N = 15–30 FRAP traces across each condition from three independent trials; data are represented as mean ± SEM. Dotted circles in (A, E, and F) indicate the bleached area in the FRAP experiments. HaloTag-fusion proteins were labeled with JF549.

We next characterized the full-length (fl) versions of each syt7 splice variant. Each variant was fused to a C-terminal HaloTag (labeled with JF549) and overexpressed in HEK293T cells (Fig. 2A, fig. S6A,B) and rat hippocampal neurons (Fig. 2E,F, fig. S7A,B). In both cases, the fusion proteins were largely targeted to the plasma membrane, where they were subjected to FRAP experiments. In HEK cells, α- and β-syt7-fl recovered 71% and 69%, respectively (Fig. 2B,C), and the t_1/2_ values are provided in table S2. In the presence of 10% 1,6-HD, recovery of both α- and β-syt7-fl was <26% (Fig. 2A–C, fig. S6A), indicating that these proteins were unable to reassemble into LLPS-mediated patches. Analogous to the *in vitro* FRAP experiments of γ-syt7cyto aggregates in Fig. 1G,H, in cells, γ-syt7-fl recovered only 21% of the signal under control conditions, while no significant changes were observed with 1,6-HD treatment (Fig. 2D, fig S6B; % recovery = 15, t_1/2_ values are provided in table S2), consistent with the conclusion that this variant does not form liquid-like dynamic assemblies.

Extending these observations to rat hippocampal neurons, the FRAP profiles of α- (Fig. 2E,F), β- (fig. S7A), and γ-syt7-fl (fig. S7B) were similar to HEK cells only within interbouton segments (Fig. 2G–I; % recovery under control and 1,6-HD conditions, respectively, for α: 78, 17, β: 89, 11, and γ: 14, 13; the t_1/2_ values are provided in table S3). These data are consistent with previous observations that syt1 and other presynaptic proteins form clusters on the plasma membrane of neurons (Willig et al., 2006). Surprisingly, the extent of recovery was severely hindered in boutons, and no significant changes were observed upon treatment with 1,6-HD (Fig. 2J–L; % recovery under control and 1,6-HD conditions, respectively, for α: 10, 17; β: 12, 12; and γ: 34, 18; the t_1/2_ values, are provided in table S3), revealing limited diffusion within boutons (see Discussion). We also conducted FRAP experiments of the α variant in the soma, dendrites, and the axon initial segment (AIS; fig. S8A–D), and observed limited recovery in all three locations, and little to no effect of 1,6-HD (fig. S8A,B,D). Together, these results demonstrate that alternative splicing and subcellular localization alter the extent and kinetics of recovery of syt7 after photobleaching.

## Alternative splicing of syt7 affects paired-pulse facilitation

**Figure 3.**
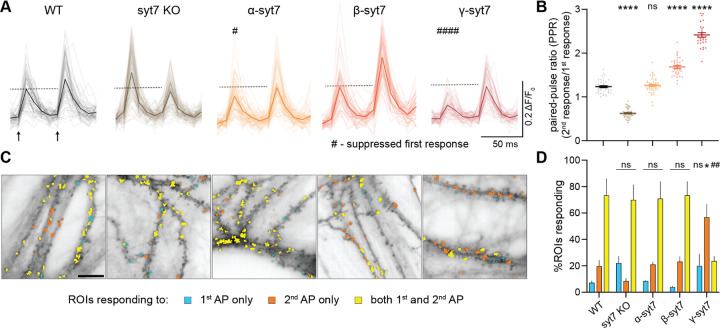
Alternative splicing of syt7 regulates paired-pulse facilitation (**A**) Representative traces of super-folder iGluSnFR.S72A (henceforth referred to as iGluSnFR) responses (ΔF/F_0_) from a field of view (FOV) of mouse hippocampal neurons after two stimuli at 20 Hz (indicated by two arrows); the imaging frequency was 100 Hz. Average traces from a FOV of wild-type (WT), syt7 KO, and syt7 KO transduced with equal amounts of α-, β-, or γ-syt7fl are in black, brown, yellow, orange, and red, respectively, with lighter shades representing traces from individual regions of interest (ROIs). The dashed line corresponds to the peak of the first response in WT condition. #*P* < 0.05; ####*P* < 0.0001 indicates suppressed first response. (**B**) Quantification of paired-pulse ratios (PPR) of peak iGluSnFR responses for all five conditions tested in (A). Note that the first response of γ-syt7 is strongly suppressed as compared to WT, and hence, PPR appears synthetically high. (**C**) Representative FOV of all five conditions from panel (A) showcasing ROIs that respond to only first (cyan), only second (orange), and both (yellow) APs. Note: the number of ROIs that respond to both the first and second APs are significantly lower in the γ-syt7 condition compared to any other condition tested, owing in part to an increased failure rate during the first AP. Scale bar, 10 μm. (**D**) Quantification of (C), demonstrating % of ROIs responding to only the first, second, or both APs. Note that α-syt7 protein expression was functionally matched to WT levels to elicit a similar PPR, and β-, and γ-syt7 expression levels were kept similar to α-syt7 (fig. S9A–C). Number of FOVs analyzed: 44, 48, 48, 35, and 30 for WT, syt7 KO, α-, β-, or γ-syt7 conditions, respectively, across three or more independent culture preparations; each FOV contains 50–200 ROIs; data are represented as mean ± SEM. To determine statistical significance, one-way analysis of variance (ANOVA) with Tukey’s correction for multiple comparisons was used in (B); two-way ANOVA with Tukey’s correction for multiple comparisons in (D). ns, not significant; *P < 0.05; ****P < 0.0001; ^##^*P* < 0.01. Comparisons to the WT condition are indicated in (B); comparisons to the WT condition for first, second, and both %ROIs responding in (D); full statistics are provided in Data S1.

To determine whether alternative splicing regulates the function of syt7, we examined presynaptic aspects of PPF and synaptic depression in cultured hippocampal neurons using iGluSnFR, an optical sensor that directly reports glutamate release (Marvin et al., 2018). After transducing neurons with a lentivirus to express the sensor, we examined PPF by triggering two APs at 20 Hz (50 ms interstimulus interval (ISI)) and calculated the ratio of the second to the first iGluSnFR response (Fig. 3A). As observed previously (Jackman et al., 2016; Turecek et al., 2017; Vevea et al., 2021; Wu et al., 2024), cultured WT mouse hippocampal neurons exhibited a paired-pulse ratio (PPR) of 1.24 ± 0.02, whereas syt7 KO neurons exhibited paired-pulse depression, with a ratio of 0.63 ± 0.02 (see Fig. 3A,B for raw traces and quantification, respectively). We then expressed each of the three splice variants in a syt7 KO background; α-syt7 was expressed at a level that functionally matched WT PPF, while β- and γ-syt7 were matched to the α-syt7 protein levels (fig. S9A–C for Western blots). Interestingly, all three variants, when expressed at equal levels, rescued PPF (Fig. 3A,B), albeit to different degrees and via different mechanisms. The β variant yielded a strong second response, even greater than in WT, with a normal first response, to enhance PPF (ratio: 1.69 ± 0.03; fig. S10A,B, Fig. 3A,B). In contrast, both the α and γ variants suppressed the first response, with γ having a particularly dramatic effect (fig. S10A). This suppression is reduced in the second response, resulting in PPF (ratios, α: 1.27 ± 0.02, γ: 2.42 ± 0.05; Fig. 3A,B, fig. S10B). These findings demonstrate clear functional differences between the three alternatively spliced isoforms of syt7.

To investigate the suppression of the first response by the α and γ variants, we leveraged the spatial readout afforded by iGluSnFR to map the regions of interest (ROIs) responding to only the first (blue), only the second (orange), or both APs (yellow; Fig. 3C). This analysis showed that γ-syt7 is, again, unusual; this variant yielded a significant reduction in the fraction of synapses that responded to both APs, as compared to all the other conditions. In addition, a significant population of synapses were recruited only upon the second AP (Fig. 3C,D). In contrast, neurons expressing α- or β-syt7 displayed a similar number of synapses that responded to both APs.

Delving further, we analyzed the PPR by examining the ROIs that only responded to both APs. This enabled us to address whether syt7 variants affect PPF by increasing glutamate release, independent of synapse recruitment during the second AP. In WT neurons, as well as KO neurons expressing each of the three syt7 splice variants equally, there was an increase in glutamate release in response to the second stimulus (fig. S11A; note: the number of synapses were the same across splice variants (fig. S12A,B)). These findings reveal that syt7-mediated PPF involves, to some degree, strengthening the output of boutons. Future work, marking all synapses, is needed to quantitatively address the contribution of synaptic recruitment.

Next, to confirm these findings and to determine how alternative splicing of syt7 regulates postsynaptic aspects of PPF, we performed whole-cell voltage clamp electrophysiology experiments on cultured hippocampal neurons and measured evoked excitatory postsynaptic currents (EPSCs) in response to two APs at a frequency range of 5–20 Hz (50–200 ms ISI; fig. S13A,B). We investigated all five conditions tested above: WT, syt7 KO, and syt7 KO neurons expressing each of the three syt7 isoforms equally, and confirmed our presynaptic glutamate release findings that both α and, more profoundly, β rescued PPF under all ISI tested (fig. S13A,B for raw traces at 20 Hz and quantification of PPR as a function of ISI, respectively). Surprisingly, our electrophysiological interrogation (fig. S13A–D) of the γ variant differed from the iGluSnFR measurements (Fig. 3A,B). Namely, γ rescued PPF, but only to the same extent as the β isoform, and without significantly suppressing the first response (fig. S13A–C). While the reasons for this disparity regarding the γ variant are currently unclear, the whole-cell voltage clamp and iGluSnFR data demonstrate that alternative splicing of syt7 regulates PPF.

## Alternative splicing of syt7 impacts synaptic depression

**Figure 4.**
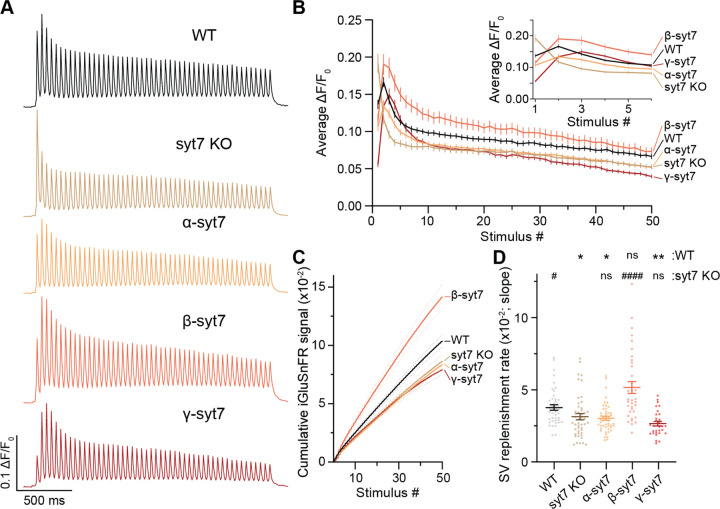
Alternative splicing of syt7 differentially regulates resistance to synaptic depression and synaptic vesicle replenishment rate (**A**) Average iGluSnFR responses (ΔF/F_0_) from mouse hippocampal neurons during high frequency stimulation (HFS; 50 action potentials at 20Hz) for WT, syt7 KO, and syt7 KO that expressed equal levels of α-, β-, or γ-syt7-fl, represented in black, brown, yellow, orange, and red, respectively. (**B**) Average amplitude of iGluSnFR responses (ΔF/F_0_) after HFS in (A). Inset shows the first six responses for all conditions. (**C**) Average cumulative iGluSnFR signal for all five conditions plotted versus stimulus number. Dotted lines represent SEM. β-syt7-fl enhances while α- and γ-syt7-fl fail to rescue total glutamate release. (**D**) Synaptic vesicle replenishment rates (SVRR) were calculated from the slope of the linear fit of the last 30 APs of the cumulative release plot (C). Number of FOVs analyzed: 44, 48, 48, 35, and 30 for WT, syt7 KO, α-, β-, or γ-syt7 conditions, respectively, across three or more independent culture preparations; each FOV contains 50–200 ROIs; data are represented as mean ± SEM. To determine statistical significance, one-way analysis of variance (ANOVA) with Kruskal-Wallis test with Dunn’s multiple comparison correction in (D). ns, not significant; *P < 0.05; **P < 0.01; ****P < 0.0001, #*P* < 0.05; ###*P* < 0.001. Comparisons in (D) are with the WT (*) and syt7 KO (#) conditions; full statistics are provided in Data S1.

Loss of syt7 accelerates and deepens synaptic depression, due to a reduction in the SV replenishment rate (SVRR) (Liu et al., 2014; Vevea et al., 2021). In the next series of experiments, we addressed the roles of the three splice variants in this form of STP. We again turned to iGluSnFR to directly measure presynaptic aspects of depression, but now during train stimulation (50 APs at 20 Hz). Average iGluSnFR traces and amplitudes are shown in Fig. 4A–B, confirming faster and greater depression during the train in syt7 KOs as compared to WT (Jackman et al., 2016; Turecek et al., 2017; Vevea et al., 2021; Wu et al., 2024). Strikingly, only the β variant rescued the depression phenotype. Of note, neurons expressing this isoform performed even better than WT, with larger amplitude glutamate signals that resisted depression (Fig. 4A–B). Neurons expressing α were largely indistinguishable from the KO; γ was similar to α but had slightly enhanced depression (Fig. 4A–B). We then plotted the cumulative iGluSnFR signal (Fig. 4C) and calculated the SVRR from the slope of a linear curve fitted to the final 30 APs of the train (Fig. 4D). Only β-syt7 was able to rescue SVRR as compared to the WT condition, while α and γ were indistinguishable from KO (Fig. 4D). In summary, under similar expression levels of the three syt7 splice variants in cultured neurons, only the β splice variant rescues both the PPF and depression phenotypes in the KO, while all three variants rescue PPF (albeit via somewhat different mechanisms). Hence, alternative splicing uncouples the roles of syt7 in these two forms of short-term plasticity.

## Two-color MINFLUX super resolution imaging reveals nanometer-scale organization of syt7 clusters at the active zone

**Figure 5.**
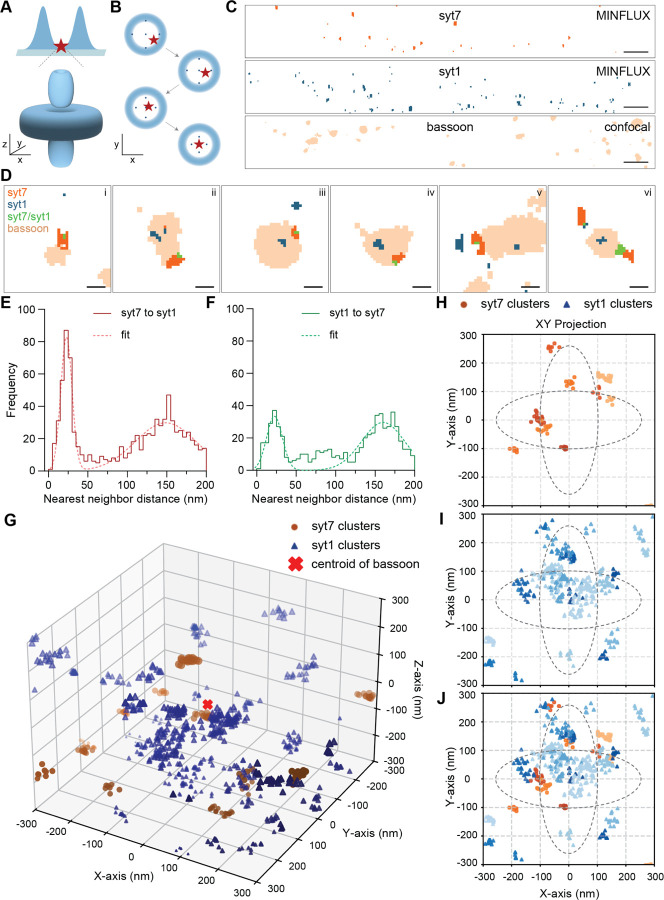
Two-color MINFLUX super-resolution microscopy reveals nanometer-scale organization of syt7 and syt1 at boutons in hippocampal neurons. (**A**) Illustration of a fluorophore (indicated as a red star) excited by a 3D donut beam (or bottle-beam shape; in blue) in minimal photon flux (MINFLUX) super resolution microscopy. (**B**) 2D schematic of the localization of a fluorophore by a 3D excitation beam progressively narrowing the probing pattern (illustrated by small blue dots) to precisely localize the fluorescent molecule. (**C**) Representative images of maximum Z-projection of syt7 (orange, MINFLUX), syt1 (blue, MINFLUX), and bassoon (tan, confocal) from a FOV. Scale bar, 2 μm. (**D**) Zoomed-in merged images (i – vi; syt7, syt1, and bassoon) from (C); overlap of syt7 and syt1 is represented in green. Scale bar, 0.2 μm. (**E,F**) Histogram of nearest neighbor distances (NND) of syt7 to syt1, and syt1 to syt7, respectively. Both graphs showed a peak around 22 nm, with the syt7 to syt1 peak being ~2.5 times larger than the syt1 to syt7 NND peak. The broad peak, centered around ~150 nm in both plots, occurs for any two random fluorescent molecules imaged by MINFLUX. The data were well-fitted with two Gaussian functions (dotted lines). (**G**) 3D representation of syt7 (orange circles) and syt1 (blue triangles) clusters distributed around the bassoon centroid (red cross). Darker shades of color indicate positive values along the X and Y axes, and larger symbols indicate positive values along the Z axis. (**H-J**) 2D scatter plot generated from the 3D graph in (G), showing syt7 clusters, syt1 clusters, and their overlap projected along the XY axes, respectively. Projections along the XZ, and YZ axes are shown in fig. S17A–C. Protein clusters were grouped using different shades. Center (0,0) indicates the bassoon centroid obtained from confocal imaging. Dotted ellipses indicate the average size of an active zone, with major and minor axes of ~500 nm and ~200 nm, respectively. N=15 FOVs, across six independent neuronal cultures.

For syt7 to directly mediate STP by controlling SV dynamics, it must be present in active zones, where release occurs. We next examined the localization of syt7, by focusing on the α variant, which is thought to be the most abundant isoform (Fukuda et al., 2002b), using super resolution imaging. Airyscan confocal microscopy (~120 nm resolution) showed colocalization of overexpressed α-syt7-fl-FLAG (referred to below as syt7), endogenous syt1, and endogenous bassoon (a marker for synaptic boutons) proteins (fig. S14A); syt7 was tagged to circumvent the lack of highly specific antibodies. To achieve nanometer-scale resolution, we utilized 3D minimal photon flux (MINFLUX) super resolution imaging, capable of resolving individual fluorophores at distances of 2–3 nm (Balzarotti et al., 2017; Grabner et al., 2022; Schmidt et al., 2021). MINFLUX precisely controls the bottle-beam-shaped excitation and achieves nanometer-level resolution through an iterative process, progressively narrowing the probing pattern, to pinpoint the location of fluorescent molecules (Fig. 5A,B). Here, we harnessed a stochastic switching strategy of fluorophores (Abberior FLUX 640 and 680 secondary antibodies for labeling primary syt1 and FLAG antibodies to detect syt1 and α-syt7fl-FLAG), which transitions between fluorescent and non-fluorescent stages using reducing buffer conditions, as detailed in the Methods. Due to the complexity of these experiments, MINFLUX imaging of the β and γ variants will be addressed in future studies, in order to determine whether changes in their linker segments affect their nanoscale localization at the active zone.

We successfully resolved 2-color 3D MINFLUX of syt7 (orange) and syt1 (blue), and achieved a fluorophore resolution (nm) of 5.39 ± 0.26, 5.14 ± 0.26, and 3.01 ± 0.22 along the X, Y, and Z axes, respectively (Fig. 5C and fig. S15A,C,D). However, the IgGs used to detect syt7 and syt1 have a linkage error of ~15 nm, so the practical resolution, which is the sum of this linkage error plus the instrument resolution, was ~21 nm in the X-Y coordinate. In parallel, we also performed confocal imaging of bassoon (tan) (Fig. 5C). Representative colocalization images of syt7 and syt1 (MINFLUX) with bassoon (confocal) are shown in Fig. 5D. As a negative control, non-transduced syt7 KO neurons, stained with the anti-FLAG antibody, exhibited a minimal background signal (fig. S16A). We also note that the syt1 IgG (DSHB, mAB48) has been extensively characterized and does not yield significant background signals using syt1 KO neurons (Courtney et al., 2021, 2019; Watson et al., 2023).

After filtering the 3D data based on localization intensity (fig. S15B,C), and combining data across multiple experiments, we generated frequency distribution plots of the nearest neighbor distances (NND) of syt7 to syt1 and syt1 to syt7, where two peaks are apparent (red and green, respectively, Fig. 5E,F, fig. S15D,E); the dotted curve represents two peaks fitted with Gaussian functions. The small NND peak was at ~22 nm, and the larger one was centered at ~150–160 nm (see table S4). The small peak was significantly larger when measuring the distance from syt7 to the nearest syt1 molecule. This suggests that syt1 and syt7 form separate groups, but a few syt1 molecules are consistently clustered very close to the syt7 group. We note that 20% of syt1 is localized to the neuronal surface, as shown by stimulated emission depletion (STED) microscopy, and pHlourin-syt1 imaging (Fernández-Alfonso et al., 2006; Willig et al., 2006), yielding a ~20% error in our NND calculations.

Next, we sought to localize syt7 and syt1, with respect to a bouton centroid, as determined via confocal images of bassoon. Briefly, we found the relative vector distances of syt7 and syt1 in X, Y, and Z coordinates from bouton centers, and combined multiple bouton centers from multiple images. The data were further processed to find syt7 and syt1 clusters using the DBSCAN algorithm, and presented as a 3D graph (Fig. 5G, see Methods section). This plot shows orange circles and blue triangles to indicate syt7 and syt1 clusters, respectively, while a red cross at (0,0,0) denotes the bouton center, which is the bassoon centroid. The graph is coded such that positive X-Y coordinates are indicated by darker color shades, while positive Z coordinates are denoted by larger symbols. To gain insights into the distribution of syt7 and syt1 around the bassoon center, we plotted 2D projections in XY, XZ, and YZ axes of syt7 (orange circles), syt1 (blue triangles), and both syt7 and syt1 (XY: Fig. 5H–J; XZ, YZ: fig. S17A–C). Dotted black lines indicate the average size of the active zone (major and minor axes diameters of ~500 nm and ~200 nm; Fig. 5H–J), isotropically arranged in the XY projection, estimated from electron micrographs and super resolution imaging (Dani et al., 2010; Schikorski and Stevens, 1997). Interestingly, syt7 clusters were mainly found at the boundary of the active zone, while no such pattern was observed for syt1. The distribution pattern of syt7 indicates that eleven out of twelve clusters were found within the active zone boundary in the 2D projection, while one was found at the periphery (Fig. 5H). In the case of syt1 clusters, which are present on SVs (~80%) and the axonal plasma membrane (~20%), we found overlap with the center of the synaptic bouton (at (0, 0) coordinate), and at the edges of the active zone (Fig. 5I). This suggests that syt1 is mainly found at the center of boutons in SV pools, while a ~20–30% proportion of these clusters were found near the active zone. A merged map of the syt7 and syt1 clusters is shown in Fig. 5J, further illustrating their partial colocalization. Collectively, these findings establish that α-syt7 is mainly found within the boundary of the active zone and α-syt7 and syt1 form distinct clusters, with a proportion of syt1 localizing with syt7 clusters.

## Discussion

Syt7 is a widely expressed (Li et al., 1995), high-affinity calcium sensor crucial for STP (Huson and Regehr, 2020). Genebank data and early studies suggested that *syt7* undergoes alternative splicing at the jxm linker (Craxton and Goedert, 1999; Fukuda et al., 2002b; Sugita et al., 2001), potentially existing as twelve different isoforms in humans (NCBI Gene ID: 9066). Fukuda et al. established the expression of three splice variants of syt7 in mice: α, β, and γ, so these are the isoforms investigated in the current study (Fukuda et al., 2002b). Here, we demonstrate that alternative splicing of syt7 regulates its self-association and show that these properties correlate with differences in syt7-mediated STP.

Firstly, we discovered that the α and β isoforms of syt7 both underwent liquid-liquid phase separation (LLPS) (Fig. 1D–F), with the β variant showing a slightly higher propensity to form droplets than α (fig. S1A,B). In stark contrast, the γ isoform formed insoluble aggregates (Fig. 1D,G). This difference was conserved in cellular environments; namely, on the plasma membrane of HEK293T cells (Fig. 2A–D, fig. S6A,B) and in the interbouton regions of neurons (Fig. 2E,G–I, fig. S7A,B). The α and β isoforms formed clusters that recovered rapidly after photobleaching (Fig. 2A–C,E,G,H, fig. S7A), whereas the γ isoform showed limited recovery (Fig. 2D,I, fig. S7B). The α- and β-syt7 patches were sensitive to 1,6-HD, consistent with assembly through LLPS (Fig. 2A–I, figs. S6A, S7A). Strikingly, none of the isoforms recovered from photobleaching within presynaptic boutons (Fig. 2F,J–L, fig. S7A,B), suggesting that syt7 is stably anchored, likely through interactions with the presynaptic cytoskeleton (Nelson et al., 2013). Thus, the diffusion of syt7 depends on both alternative splicing and subcellular localization in hippocampal neurons.

The founding member of the syt family, syt1, was also recently shown to undergo LLPS (Mehta et al., 2024). Both isoforms, syt1 and syt7, sense Ca^2+^ (Bhalla et al., 2005), but the former is a fast, low-affinity sensor (Hui et al., 2005) that triggers SV exocytosis (Bai et al., 2016; Courtney et al., 2019; Evans et al., 2015; Geppert et al., 1994; Littleton et al., 1993), while the latter is a slow, high-affinity sensor (Hui et al., 2005) that regulates transient SV docking (Wu et al., 2024). In the case of syt1, LLPS separation was shown to play a crucial role in clamping spontaneous SV release, as well as triggering rapid, efficient evoked exocytosis (Courtney et al., 2021). Droplets formed by syt1, α-syt7, and β-syt7 recover from FRAP to similar extents (Fig. 1H), and the cytoplasmic domains of all three proteins formed droplets in neurons (fig. S4A,B) (Mehta et al., 2024). However, Ca^2+^ promoted syt1 droplet formation (Mehta et al., 2024) but had no effect on syt7 droplets (Fig. 1K). The basis for this difference is unclear, but it suggests a form of regulation of syt1 that does not occur with syt7. Rather, syt7 LLPS is regulated by alternative splicing.

The isoform-specific biophysical differences regarding LLPS exhibited by the syt7 splice variants were correlated with distinct functional outcomes for synaptic transmission. Presynaptic function was monitored using iGluSnFR and revealed that the β isoform is a “gain-of-function” variant; it enhanced PPF relative to the α isoform (Fig. 3A,B), without any changes in the first response (fig. S10A). Moreover, β rescued the SVRR (Fig. 4C,D) while counteracting synaptic depression more effectively than any of the other rescue conditions, and even better than WT neurons (Fig. 4A,B). Surprisingly, γ, when expressed at the same level as α and β, failed to rescue the SVRR or synaptic depression phenotypes (Fig. 4A–D), yet gave rise to the highest PPF ratio (Fig. 3A,B), with a caveat: it suppressed the initial response (fig. S10A), by decreasing the number of synapses that responded to the first AP (Fig. 3C,D). This shift in release probability (P_r_) does not appear to significantly change in any other condition except α, which slightly reduced the magnitude of the first response as compared to WT (Fig. 3A,B, fig. S10A). The α variant also enhanced the amplitude of the second response to rescue PPF to WT levels (Fig. 3A,B, fig. S10B), but, like γ, α failed to rescue the SVRR or synaptic depression phenotypes in the KO (Fig. 4A–D). In the context of the α and γ variants findings, we note that syt7 KO neurons do trend toward increased P_r_ (Fig. 3A,B, fig. S10A) (Vevea et al., 2021; Wu et al., 2024). While this trend has not reached significance in the current (fig. S10A) or previous studies (Vevea et al., 2021; Wu et al., 2024), it suggests that splice variants that reduce the first response are likely to be expressed at low levels in hippocampal neurons.

To examine postsynaptic responses, we conducted whole-cell voltage clamp recordings and observed rescue of PPF by both α- and β-syt7, with β mediating even greater facilitation than WT neurons. These findings are congruent with the iGluSnFR measurements (fig. S13A,B). However, the γ variant differed: via iGluSnFR, γ yielded even higher PPF than β, but when measured using post-synaptic voltage clamp recordings, neurons expressing the γ and β variants had similar PPF values. As noted above, the reasons for this difference are unclear; however, we argue that iGluSnFR provides a reliable measure of the absolute level of glutamate release, whereas voltage clamp recordings provide a somewhat relative measure of transmission. More specifically, in the latter approach, we determine the stimulus intensity needed to reliably trigger ~200 pA of post-synaptic α-amino-3-hydroxy-5-methyl-4-isoxazolepropionic acid (AMPA) receptor current (fig. S13C), and this differed to some extent across conditions, with the γ condition requiring the greatest depolarizing current (fig. S13D). In contrast, for iGluSnFR, the same field stimulation, a platinum wire that depolarizes all the neurons on the cover slip, is used in all experiments. These differences may explain, in part, why the first response is sharply suppressed in neurons expressing γ when measured via iGluSnFR (Fig. 3A,B, fig. S10A). These findings indicate that it will be important to continue to compare presynaptic release with post-synaptic responses to better understand the relationship between secretion and transmission. For example, it remains possible that significant levels of glutamate release might not be detected via post-synaptic recordings.

For syt7 to function presynaptically to control STP, we sought to determine whether it is present at the active zone, where SVs dock and fuse. Using MINFLUX super resolution imaging, we localized syt7 to clusters within the active zone boundary (Fig. 5H). Some of these clusters overlapped with syt1 clusters (Fig. 5I,J), consistent with syt1-bearing SVs colocalizing with syt7 at the active zone (fig. S18A). Hence, syt7 is well-positioned to regulate STP by controlling aspects of the SV, and in particular, the docking step, as discussed below (Vevea et al., 2021; Wu et al., 2024).

We emphasize that syt7 is not tailored to trigger SV release but rather modulates synaptic strength in response to recent activity. Owing to its higher Ca^2+^ affinity and slower kinetics, syt7 is particularly sensitive to residual Ca^2+^ (Jackman and Regehr, 2017), which lingers in the presynaptic terminal after an AP. Recently, syt7 has been proposed to regulate transmission by mediating activity-dependent “transient” SV docking (Vevea et al., 2021; Wu et al., 2024). We further explored this idea and interrogated whether SVs bind directly to syt7. Indeed, HaloTag-pull down assays indicated an interaction between α-syt7cyto and purified SVs, and this interaction was weakly promoted by Ca^2+^ (Ca^2+^/EGTA condition: 1.34 ± 0.23; fig. S19A,B). We further addressed this docking hypothesis by exploring binding partners of syt7 via immunoprecipitation (IP; fig. S19C,D) using rat hippocampal neurons expressing α-syt7-fl-FLAG. In the absence of cross-linkers, we identified a sole and specific interaction between syt7 with syt1 (fig. S19D), as observed previously (Fukuda and Mikoshiba, 2000). We validated these findings using immobilized syt7 and syt1-fl that had been reconstituted into liposomes and, again, observed an interaction that is modestly enhanced by Ca^2+^ (Ca^2+^/EGTA: 1.46 ± 0.15; fig. S19A,B). It is likely that the weak Ca^2+^-dependence is mediated by the C2-domains of one or both of these proteins, but, interestingly, we also found that the jxm linkers of syt7 and syt1 directly bind to one another in a concentration-dependent manner (fig. S19E,F). Moreover, these jxm linker segments mediate LLPS, and α-syt7cyto and syt1cyto droplets coalesce *in vitro* (fig. S20A) and in neurons (fig. S20B), further confirming their interaction.

Together, the findings reported here are consistent with a model in which syt7 is a docking protein that mediates PPF (Vevea et al., 2021; Wu et al., 2024). After a single AP, ~40% of all SVs undock (Kusick et al., 2020), and syt7 is crucial for rapidly re-docking vesicles to refill the “slots” for the next round of release (Wu et al., 2024), potentially mediated, in part, by coalescence of syt7-syt1 phase-separated surfaces. This would occur during activity, as – again – docking is unaffected under resting conditions in syt7 KO neurons (Wu et al., 2024), and other known protein and lipid interactions have been shown to participate in the attachment of SVs to the active zone membrane (Mochida, 2021; Verhage and Sørensen, 2008). This 2D LLPS interaction, mediated by the syt7 and syt1 jxm linkers (figs. S19E,F, S20A,B), might juxtapose their C2-domains to enhance Ca^2+^-promoted binding between them, thus recruiting more SVs to release sites during ongoing activity. Surprisingly, iGluSnFR measurements revealed that only the β splice variant was able to rescue the SVRR and synaptic depression phenotypes (Fig. 4A–D), while α and γ failed, despite the finding that all three rescued PPF (Fig. 3A,B). Thus, alternative splicing can uncouple the role of syt7 in these two different forms of short-term plasticity. While the leading model for syt7 function in PPF is via activity-dependent docking, the findings reported here reveal that the role of syt7 during synaptic depression is more complex, involving either additional or completely different mechanisms. Hence, the differences in function of α, β, and γ provide a molecular handle to delve further into the mechanisms that underlie STP. Future zap-and-freeze electron microscopy studies of neurons expressing each splice variant will shed light on the transient docking question. Another important goal is to purify the cytoplasmic domains of all three variants in non-droplet and non-aggregated forms to enable biochemical studies to investigate how the jxm linkers affect Ca^2+^ and effector binding.

In summary, the current study demonstrates that alternative splicing of the syt7 jxm linker serves as a molecular switch, toggling the protein between dynamic, functional condensates and static, potentially disruptive aggregates. These properties correlated, to some extent, with a functional impact on synaptic plasticity and might even play a role in determining the initial P_r_. Since alternative splicing affects only the linker domain of syt7, it seems likely that differences in linker-mediated self-association underlie the observed differences in function. It will be important to delve deeper into this issue via mutational analysis and linker-swapping experiments, to further establish causation. A particularly challenging issue is to understand how subtle differences in the propensity to phase separate, between the α and β variants, translate to changes in PPF, and to determine how the γ variant, which forms aggregates, is able to sharply suppress the fraction of active synapses responding to a stimulus. In this context, it is notable that the *Drosophila* ortholog of syt7 was reported to be inhibitory (Guan et al., 2020), but whether this ortholog phase separates or aggregates has yet to be determined. Given that protein condensation and aggregation are inherently concentration-dependent phenomena, a crucial future direction will be to investigate how lower expression levels of the γ-variant affect its biophysical and functional properties. Furthermore, protein aggregation is a recognized hallmark of several neurodegenerative diseases (Ross and Poirier, 2004), so dysregulation of the γ variant can potentially contribute to pathophysiology. Finally, while this study focused on the α, β, and γ isoforms of syt7, it is unclear which mixture of variants is expressed at which synapses. Moreover, we recognize that there may be other, undetected syt7 isoforms that are expressed in the brain (NCBI Gene ID: 9066). Spatially resolved transcriptomics are now needed to address this issue. This new direction is crucial, as the three splice variants characterized thus far, as shown here, have distinct biophysical and functional properties. Finally, we note that among the seventeen isoforms of syt (not including splice variants), ten isoforms possess IDRs within their jxm linkers. It will be interesting to determine which isoforms undergo LLPS, and to address how phase separation impacts their function.

## Material and Methods

### DNA constructs

Intensity-based glutamate-sensing fluorescent reporter (iGluSnFR) S72A was a gift from Looger L. (Janelia Farm, Ashburn, VA; AddGene ID# 106176) (Marvin et al., 2018), but was modified with CAMKIIα promoter as previously described (Vevea et al., 2021). α-syt7 cDNA was a gift from Fukuda M. (Tohoku Neuroscience Global COE, Sendai, Japan) (Fukuda et al., 2002b). β- and γ-syt7 sequences were ordered as geneblocks from Integrated DNA Technologies; all three splice variants α, β, and γ-syt7-full length (fl) were assembled using PCR splicing with overlap extension and subcloned into transfer plasmid FUGW with human synapsin (hSyn) promoter and WPRE (Woodchuck hepatitis virus posttranscriptional regulatory element) 3’ untranslated region (UTR) element. FUGW was a gift from Baltimore D. (California Institute of Technology, Pasadena, CA; AddGene plasmid #14883) (Lois et al., 2002). pLenti-hSyn-CRE-WPRE plasmid was used for CRE expression (a gift from Fan Wang; Duke University Medical Center, Durham, NC) (Sakurai et al., 2016). Constructs encoding the complete cytoplasmic domains of the alternative splice variants of syt7 were modified to add an N-terminal 6XHis tag, and a C-terminal fluorescent tag, mRuby3 (referred to as mRuby in the text). These fusion constructs were subcloned into the pET28(a)+ plasmid for bacterial expression. Rat syt1 cDNA was provided by T. C. Sudhof (Stanford University, Stanford, CA) (Perin et al., 1990); the D374 mutation was corrected by replacement with a glycine. Constructs encoding SUMO-syt1-fl, syt1 cytoplasmic domain fused with GFP, and syt1 juxtamembrane linker fused with GFP were subcloned into pET28(a)+ plasmid for bacterial expression.

### Recombinant protein expression

Recombinant proteins were expressed as described previously (Mehta et al., 2024). Briefly, all recombinant proteins were expressed in *E. coli* BL21(DE3) (NEB, C2527H) cells at 37°C until an OD of 0.6 was achieved. Bacteria were then induced with 500 μM isopropyl β-D-1-thioga-lactopyranoside (IPTG) (GoldBio, I2481C) followed by overnight growth at 18°C. Cells were harvested and lysed using sonication, followed by solubilization with 1% Triton TX-100 (Thermo Fisher Scientific, A16046) for 2 h. A two-step protein purification, involving affinity and size-exclusion chromatography, was performed using Cobalt TALON affinity resin (Takara, 635653) and a Superdex 200 Increase 10/300 GL column (Cytiva, 28990944) on a fast protein liquid chromatography instrument (FPLC; Akta). Proteins were eluted in 25 mM Tris pH 7.4 buffer with 500 mM NaCl. To minimize proteolytic activity, a protease inhibitor cocktail (PIC) (Roche, 046693132001) was used during the lysis steps. High salt washes (0.5–1 M NaCl) were performed to remove any contaminants. For syt1-fl protein purification, similar steps plus 0.9% CHAPS detergent (3-((3-cholamidopropyl) dimethylammonio)-1-propanesulfonate; Fisher Scientific, 50-223-7023) were added during lysis and maintained throughout the purification steps. Syt1-fl was cleaved from the beads with SUMO protease treatment. Finally, purified proteins were subjected to SDS-PAGE, and protein concentration was determined using bovine serum albumin (BSA) (Jackson ImmunoResearch, 001-000-162) as a standard.

### Self-association assay

*In vitro* self-association assays were conducted as described previously (Mehta et al., 2024). In short, mRuby-fused proteins (Fig. 1D, fig. S1A–F) were assessed for self-association in 25 mM Tris pH 7.4, 100 mM NaCl buffer conditions, at varying [protein] and [PEG 8000] (Sigma, P2139). In Fig. 1J,K, [NaCl], and [Ca^2+^] were varied. Protein droplets and aggregates were imaged using a ZeissAxioVert.AX10 and Zeiss 880 Airyscan LSM microscope with a 63X/1.4 NA oil objective at room temperature (RT). Ten μl of each sample was placed on an 18 mm coverslip (Warner Instruments, 64–0734, CS-18R17), and the settled droplets and aggregates were imaged. Images were analyzed using the threshold and analyze functions in Fiji. Data were plotted using GraphPad Prism.

### HEK293T and neuronal cell culture

HEK293T cells (ATCC, CRL-11268) were maintained in Dulbecco’s Modified Eagle Medium (DMEM) with high glucose (Gibco, 11965092), supplemented with 10% fetal bovine serum (FBS; R&D Systems, S11550H) and penicillin-streptomycin (Thermo Fisher Scientific, MT-30–001 CI), as described previously (Mehta et al., 2024). Hippocampal neurons were isolated from pre-natal Sprague-Dawley rats (Envigo) on E18. HEK293T cells and rat neurons were plated on 18 mm coverslips (Warner instruments; 64–0734 (CS-18R17)) that had been coated with poly-D-lysine (Thermo Fisher Scientific, ICN10269491) for 1 h at RT, at a density of 100K (HEK293T cells) or 125K (rat hippocampal neurons) per coverslip, in supplemented DMEM.

Mouse hippocampal neurons from *syt7fl/fl* mice (#C57BL/6N-Syt7^em1(IMPC)H^/H; repository ID: EM:14672; Mary Lyon Centre at MRC Harwell which is the UK node of the European Mouse Mutant Archive (EMMA)) (Bradley et al., 2012; Mianné et al., 2017; Pettitt et al., 2009; Skarnes et al., 2011) maintained as homozygotes, were isolated between P0-P1. Hippocampal tissue was dissected and maintained in chilled Hibernate-A media (BrainBits; HA). Post-dissection, neuronal tissue was incubated in 0.25% Trypsin (Corning; 25–053 CI) for 30 min at 37°C, and washed 2x with DMEM supplemented with 10% FBS, and Penicillin-Streptomycin. Tissue was triturated with a 1 ml pipette tip (1 mm diameter) until homogeneous. Cells were then counted with a Scepter 3.0 automated cell counter (Millipore Sigma; PHCC340) loaded with a 40 μm tip; gated to a cell diameter of 7–12 μm, and plated in DMEM media on 18 mm glass coverslips coated with poly-D-lysine and EHS laminin (Thermo Fisher Scientific; 23017015) at a density of 250K cells per coverslip. Both rat and mouse hippocampal neurons were incubated for 1 h at 37°C, 5% CO_2_, after which DMEM media was aspirated and immediately replaced with neurobasal media-A (NBM-A, Thermo Fisher Scientific; 10888–022) supplemented with 2% B-27 (Thermo Fisher Scientific; 17504001) and 2 mM Glutamax (Gibco; 35050061). Following plating, neurons were fed with NBM-A plating media once a week.

### Transfection and transduction

Lipofectamine-based transfection (Thermo Fisher Scientific, 15338–100) was carried out as previously described (Mehta et al., 2024). Lentivirus production and transduction were performed as previously described (Vevea et al., 2021). Lentivirus that expressed CRE was added to neuronal cultures at 1 day *in vitro* (DIV), while virus for the expression of iGluSnFR S72A and syt7-isoforms was added at 5–6 DIV. All viruses were titered by either fluorescence or western blot prior to usage.

### Airyscan imaging and fluorescence recovery after photobleaching (FRAP) experiments

HEK293T cells and cultured rat hippocampal neurons were imaged in standard ECF imaging solution at 37°C and 5% CO_2_. Temperature, CO_2_, and humidity were controlled using an Okolab incubation system (Okolab, Bold Line, Italy). FRAP was carried out on protein droplets/aggregates formed *in vitro*, and in HEK293T cells and rat hippocampal neurons using the photobleaching and time series modules of a Zeiss 880 Airyscan LSM microscope with a 63X/1.4 NA oil objective, using Fast Airyscan mode at RT (*in vitro* droplets) and 37°C (cell-based experiments). Briefly, we bleached circular regions of interest (0.8–2 μm in diameter) within protein droplets (2–2.5 μm in diameter) and aggregates using 405 and 488 laser lines at 25% and 100% laser power, respectively. Imaging was performed at 15 frames per minute. Samples were monitored for 20 s, 500 ms, and 400 s during pre-bleaching, bleaching, and recovery, respectively. To test for reversible dissolution of droplets, HEK293T cells and cultured rat hippocampal neurons were treated with 10% 1,6-hexanediol (1,6-HD; Sigma, 88571) for 10 min. All images were processed with automatic Airyscan deconvolution. We normalized the fluorescence traces using the equation:

$$FRAP\left(t\right)=\frac{F.I._{bleach}\left(t\right)-F.I._{background}\left(t\right)}{F.I._{non-bleached}\left(t\right)-F.I._{background}\left(t\right)}$$

where, *F.I*. indicates fluorescence intensity. We performed FRAP experiments and averaged the *F.I*. data to obtain a single FRAP curve. Data were represented as mean ± SEM.

### Dynamic light scattering (DLS)

DLS was carried out using a DynaPro Nanostar II Dynamic Light Scattering instrument (Waters Wyatt Technology) as described previously (Mehta et al., 2024), using 10 μM protein buffered in 25 mM Tris pH 7.4, 100 mM NaCl, and 3% PEG 8000. Average diameter distributions were modeled using Rayleigh Spheres in the DYNAMICS v8 (Waters Wyatt Technology) software package. Samples were assayed in triplicate using three independent preparations, and the results were presented as mean ± SEM.

### iGluSnFR imaging and quantification

iGluSnFR imaging and quantification were performed as previously described (Vevea et al., 2021; Wu et al., 2024) using iGluSnFR S72A (Marvin et al., 2018) with minor modifications. Briefly, experiments were performed on an Olympus IX83 inverted microscope using an X-Cite 120 LED (Lumen Dynamics) and ORCA-Fusion CMOS camera (Hamamatsu Photonics). For high-frequency stimulation (HFS), neurons were depolarized with 50 action potentials (APs) at a frequency of 20 Hz (total of 2.5 s) by field stimulation, and 350 frames with 10 ms exposure at 2×2 binning were collected (total of 3.5 s). Extracellular imaging media was standard ECF with an osmolarity of ~320 mOsm. Imaging media was supplemented with 50 μM D-AP5 (Abcam, ab120003), 20 μM CNQX (Abcam; ab120044), and 100 μM Picrotoxin (Tocris; 1128) to block recurrent activity. All experiments were carried out at 33–34°C, with neurons between 14–15 DIV. Temperature and humidity were controlled by a Tokai incubation controller and chamber (Tokai Hit, PPZI3). A custom ImageJ plugin was used to identify iGluSnFR regions of interest (ROIs) (Vevea et al., 2021; Wu et al., 2024). Results from the plugin were imported to AxographX1.8.0 (Axograph Scientific), where traces were normalized to the initial 500 ms pre-stimulus baseline and corrected with a two-tailed background subtraction. Glutamate release signals were defined as fluorescence that was >4x the standard deviation (SD) of the noise. The synchronous fraction (within 10 ms of an AP) of the iGluSnFR signal was measured from multiple ROIs in each field of view (FOV). For spatial analysis, ROIs corresponding to the peaks from glutamate release signals from the 1^st^ and 2^nd^ AP were identified, color-coded, and superposed on the average FOV pre-stimulation.

### Electrophysiology recordings in cultured neurons

Whole-cell voltage clamp recordings were performed on primary hippocampal cultures between 15–17 DIV. The investigator was blind to the groups during acquisition and analysis. Neurons were visualized using an inverted microscope (Olympus, IX-71). All the chemicals were from Sigma-Aldrich unless otherwise mentioned. Recording electrodes were pulled from borosilicate glass capillaries (Sutter Instruments, BF150-110-10HP) using a horizontal puller (Sutter Instruments, P1000). The pipette resistance (Rp) was 6–8 MΩ when filled with the following in mM: 130 potassium-gluconate, 10 HEPES, 5 QX-314 (Hello Bio, HB1030), 5 di-sodium phosphocreatine hydrate, 2 magnesium-ATP, 2 EGTA, and 0.3 sodium-GTP hydrate. Recordings were obtained at RT in an external solution that had in mM: 128 NaCl, 5 KCl, 25 HEPES, 30 glucose, 1 MgCl_2_, and 2 CaCl_2_ dihydrate. Neurons were clamped at −70 mV using an amplifier (Molecular Devices, MultiClamp 700B) controlled by software (Molecular Devices, MultiClamp Commander and Clampex 11.3). Responses were filtered at 2 kHz and sampled at 10 kHz using a digitizer (Molecular Devices, Digidata 1440). GABAR (gamma-aminobutyric acid receptor) currents and NMDAR (N-methyl-D-aspartate receptor) currents were blocked by 50 μM picrotoxin (Tocris, #1128) and 50 μM APV (Hello Bio, HB0225), respectively. A concentric bipolar electrode (FHC Inc., #30204) was placed near the soma of the recording neuron (100–200 μm away) to elicit AMPAR (α-amino-3-hydroxy-5-methyl-4-isoxazolepropionic acid receptor)-mediated excitatory postsynaptic currents (EPSCs). Responses were evoked by delivering 0.6 ms square-wave pulses with an isolated pulse generator (WPI Inc., A385) in current mode, triggered by the amplifier. The stimulating electrode placement was adjusted until a clean EPSC with fixed latency and no polysynaptic activity was observed. Stimulus intensity was set to elicit an EPSC of size 200–250 pA, and it varied between the recordings (25–250 μA). Paired pulses were delivered at various interstimulus intervals (ISIs, 50–200 ms). Five sweeps were obtained at 0.1 Hz (10 s) at each ISI, and those sweeps devoid of network activity (~95%) were averaged for the analysis. Various electrophysiological parameters were measured using software (Molecular Devices, Clampfit 11.3), and the ratios were calculated by dividing the peak amplitude of the second EPSC by the first (EPSC_2_/EPSC_1_). Access resistance (Ra) was monitored using a 2 mV test pulse before evoking EPSC and calculated post-acquisition. Neurons with Ra>25 MΩ or varied by >20% from the initial values were not considered for the final analysis. The series resistance (R_s_) was not compensated.

### Immunoblotting

Immunoblotting was carried out as previously described with modifications (Vevea et al., 2021). Briefly, cell cultures were washed with 1x ice-cold PBS (Thermo Fisher scientific, 14190136) and lysed with 150 μl of lysis buffer (1x PBS, 2% SDS, 1% Triton X-100, 10 mM EDTA plus protease inhibitors: PMSF (Sigma-Aldrich, 11359061001), aprotinin (Sigma-Aldrich, A6103), leupeptin (Sigma-Aldrich, L9783), and pepstatin A (Sigma-Aldrich, P5318)). Lysed cell material was subjected to centrifugation at 20,000 x g for 30 min at 18°C, and the supernatant was collected. Lysates were then subjected to a bicinchoninic acid (BCA) assay (Thermo Fisher Scientific, 23227). After total protein quantification, a final concentration of 1x Laemmli Buffer (Bio-Rad, 1610747) plus 5% β-mercaptoethanol (BioRad, 1610710) was added to lysates, which were subsequently boiled at 100°C for 3 min. For protein detection, 5 μg of total protein was subjected to SDS-PAGE using 4–20% TGX-Stain Free gels (Bio-Rad, 5678094), and the gels were imaged on a Chemi-Doc MP system (Bio-Rad) following a 5 min activation time, and the resulting image was used as a total protein loading control. Protein gels were transferred to a PVDF membrane (EMD Millipore, IPFL00010) for 30 min per gel at constant current (240 mA), then blocked with 5% nonfat milk protein in Tris-buffered saline plus 1% Tween 20 (TBST) for 30 min.

PVDF membrane was incubated with primary antibody in 2.5% non-fat milk protein/TBST overnight. After three 10 min washes using TBST, the secondary antibody in 2.5% non-fat milk protein in TBST was incubated at RT for 1 h. After three 10 min washes with TBST, blots were incubated with a rabbit secondary antibody-HRP conjugate (BioRad, 1721019) for 1 h at RT. Blots were again washed thrice for a total of 30 min. Immunoblots were imaged using Luminata Forte Western HRP substrate (EMD Millipore; ELLUF0100) and a ChemiDoc MP Imaging System (Bio-Rad Laboratories). Bands were analyzed by densitometry, and contrast was linearly adjusted for publication using Fiji. A detailed list and sources of antibodies are included in the ‘Antibody‘ section.

### Immunocytochemistry (ICC)

Dissociated mouse hippocampal neuronal cultures were fixed with ice-cold 100% methanol, permeabilized with 0.2% saponin (Sigma Aldrich, 47036), blocked with 0.04% saponin, 10% goat serum (AbCam, ab7481), and 1% BSA in PBS, followed by immunostaining with primary antibody at 4°C overnight. Coverslips were washed with PBS three times and stained with secondary antibody in 0.1% BSA and 0.04% saponin in PBS for 1 h. Following three more PBS washes, the coverslips were mounted on microscope slides (Thermo Fisher Scientific, 22–178277), using ProLong Glass Antifade with Mountant with NucBlue Stain (Thermo Fisher Scientific, P36981), and imaged. A detailed list and sources of antibodies are included in the ‘Antibody‘ section.

### Two-color MINFLUX imaging

Minimal photon flux (MINFLUX) super-resolution imaging setup was done as previously described (Balzarotti et al., 2017; Schmidt et al., 2021). Briefly, an excitation laser beam (green) is shaped by a vortex-phase mask, forming a donut-shaped intensity spot in the focal plane of the objective lens. Photons emitted by the fluorescent molecule (star) are collected by the objective lens and directed toward a fluorescence bandpass filter and a confocal pinhole by using a dichroic mirror. Intensity modulation and deflection, as well as photon counting, are controlled by a field-programmable gate array. For sample preparation, mouse hippocampal neurons expressing α-syt7-FLAG were fixed and permeabilized as described in ICC. Syt1, FLAG-tag, and bassoon were labeled with primary (see Antibodies section) and secondary antibodies (FX640, FX680, and Alexa 488). We note that there is a linkage error of ~15 nm due to primary and secondary antibody labeling.

To stabilize the sample during measurements, gold nanoparticles (BBI Solutions, EM.GC150) were used as fiducial markers (Balzarotti et al., 2017; Schmidt et al., 2021). These nanoparticles were applied to the samples, and any unbound particles were washed away with PBS. For imaging, a buffer containing glucose oxidase, called GLOX buffer, was used (50 mM Tris-HCl pH 8.0, 10 mM NaCl, 10% (w/v) glucose, 64 μg/ml catalase, 0.4 mg/ml glucose oxidase, and 25–50 mM mercaptoethylamine (MEA)). After mounting, samples were sealed with twinsil (picodent). Images were collected using Abberior Instruments Imspector software (version 16) with MINFLUX drivers, using a 100X magnification NA 1.4 oil objective lens. Stabilization was achieved using a linearly polarized 980 nm laser (Thorlabs Inc., LP980-SF15). After selection of a FOV, 75 × 75 μm confocal images were acquired. For activation of single fluorophores, a 405 nm wavelength laser (HÜBNER Photonics, Cobolt MLD 405 nm 50 mW) whose intensity was attenuated into the nW region by a neutral-density filter. Photons emitted from the sample were counted using two avalanche photodiodes.

### MINFLUX image analysis

Data acquired from Abberior Imspector software were saved as .MSR and exported into the .NPY format. These raw data were processed to optimize EFO, CFR, and len_min with values ranging from 100–300 kHz, 0–0.9, and >3, respectively (Note: the definitions of these parameters are defined below). For spectral unmixing of the two fluorophores, DCR was fitted with two Gaussian functions to separate FX640 and FX680. The mismatch in the refractive index between the #1.5 coverglass and the aqueous imaging buffer (GLOX buffer) distorts the position measurement along the Z-coordinate. This effect makes the focal point appear shallower than it actually is. To correct for this distortion in standard samples, a correction factor of 0.7 was applied to the measured Z-position. To find the nearest neighbor distance (NND), the distances between color-coded TIDs were calculated and sorted. To obtain confocal images of bassoon, .MSR files obtained through FIJI, and the centroid of all bassoon puncta was calculated (thresholding via the Otsu method, radius range in pixels 3–20, overlap threshold 0.75, and eccentricity threshold 0.9375) (Otsu, 1979). Relative positions of every TID in the 640 and 680 DCR split were updated based on the bassoon centroids, and all the localizations were merged. Clustering was performed using density-based spatial clustering of applications with noise (DBSCAN). This algorithm groups together points that are closely packed, marking outliers as points that lie alone in low-density regions. It works by identifying “core points” which have a minimum number of neighbors (5–8 in this case) within a certain radius (22.5 nm in this case), and it expands clusters outwards from these points. Any point that is not reachable by this expansion is labeled as noise, allowing the algorithm to find arbitrarily shaped clusters and effectively handle outliers without a predefined number of clusters. After clustering, all the clusters within 300 nm of the centroid were plotted in 3D and 2D projections in XY, XZ, and YZ. All analysis was done using Python.

The relevant definitions for this section of Methods are as follows:

- Effective frequency at offset (EFO): effective emission frequency (in Hz) measured at offset pattern positions.
- Center frequency ratio (CFR): the emission frequency measured at the center position of the pattern (EFC) is divided by the emission frequency measured at the offset pattern positions (EFO). CFR = EFC / EFO.
- Detection channel ratio (DCR): The counts measured on ‘Gate Channel 1’ is divided by the counts measured on ‘Gate Channel 1’ and ‘Gate Channel 2’:
- Trace ID (TID) is trace ID. Consecutive localizations from one event have the same trace ID.
- Len_min: minimum number of localizations per tid.

### Immunoprecipitation (IP)

Magnetic Dynabeads M270 Epoxy (Thermo Fisher Scientific, 14302D) were covalently linked to the FLAG antibody (Rabbit FLAG M2 antibody, Sigma) as follows: 10 mg of beads were mixed with 250 μg of antibody in 400 μl of borate buffer (100 mm sodium borate, pH 8.5) plus an additional 200 μl of 3 M ammonium sulfate overnight at 37°C. Purified control rabbit IgG was also conjugated to Dynabeads as a negative control. After coupling, the beads were subjected to a series of six rigorous 1 ml washes with alternating pH buffers (500 mM NaCl, 50 mM ammonium acetate, pH 4.5, and 500 mM NaCl and 50 mM Tris-HCl, pH 8.0) using a magnetic stand to remove unbound antibody. Finally, the prepared antibody-coated beads were washed and resuspended at 30 mg/ml in 150 mM KCl and 50 mM Tris-HCl, pH 8.0, and stored at 4°C until needed.

Rat hippocampal neurons expressing α-syt7-fl-FLAG were lysed using lysis buffer (25 mM Tris-HCl, pH 7.4, 100 mM NaCl, 1 mM EGTA, 5% glycerol, 1% TritonX-100 along with a protease inhibitor cocktail (1 tablet/ 10 ml; Sigma Aldrich 11836170001). After solubilizing on ice for 20 min with intermittent agitation, large insoluble cell debris was removed by centrifugation (4 min, 13,000 × *g,* 4°C). Supernatant was collected (Input fraction) and incubated with FLAG-Dynabeads on ice, with rotation, for 2 h. Beads were collected using a magnetic stand, and the supernatant fraction was stored. Beads were washed thrice with the lysis buffer, followed by elution with 4x Laemmli sample buffer (Bio-Rad, 1610747) with β-mercaptoethanol (Bio-Rad, 1610710). All samples were heated at 75°C for 10 min, and subjected to SDS-PAGE and immunoblot analysis.

### Synaptic vesicle (SV) purification

SV purification was performed as described previously (Bradberry et al., 2022), again using Magnetic Dynabeads M270 Epoxy (Thermo Fisher Scientific, 14302D) conjugated to the Rho1D4 antibody (purchased from the University of British Columbia (https://ubc.flintbox.com)) as described in the IP section.

SVs were isolated from WT mice generated from crossing synaptophysin (Leube et al., 1989) and synaptogyrin (Janz et al., 1999) breeder colonies at 14 – 20 days of age and of both sexes. Mice brains were isolated and homogenized in 125 mM KCl, 20 mM potassium phosphate, 5 mM EGTA, and protease inhibitors (cOmplete Mini EDTA-free, 1 tablet/10 ml; Sigma Aldrich 11836170001), pH 7.3) to create a crude lysate; all materials throughout the isolation procedure were maintained at 4°C. This lysate was centrifuged (20 min, 35,000 × *g*, 1°C) to remove large debris. Antibody-coated Dynabeads were incubated with 1.9 ml of the brain supernatant and incubated for 25 min with rotation on ice. The beads were collected using a magnetic stand, and the supernatant was discarded. Beads were then washed four times with an ice-cold wash buffer (145 mM KCl, 10 mM potassium phosphate pH 7.3), and SVs were eluted by incubating with 50 μl of 1 mM 1D4 peptide (Cube Biotech) for 30 min on ice in a buffer containing 100 mM sodium borate pH 8.5.

### HaloTag-pull down assay

HaloLink resin was washed extensively before attachment of protein, and blocked with BSA to prevent non-specific binding. Purified Halo-tagged proteins (~1.2 mg, ‘bait’ proteins) were immobilized on HaloLink resin (750 μl bed volume) for 1 h, at 25 °C, followed by 3x buffer wash. Buffer was 25 mM HEPES pH 7.4, 100 mM KCl, and indicated 0.2 mM EGTA or 1.2 mM Ca^2+^. Complete binding was confirmed by SDS-PAGE analysis of the supernatant. Prey proteins at indicated concentrations were incubated with ‘bait’-protein-tagged HaloLink bead slurry, such that the final concentration of the bait protein was 7 μM. This mixture was incubated for 1 h with rotation at 25°C and supernatants obtained by centrifugation (3500 × *g*, 10 min) were used for SDS-PAGE and Western blotting.

### Proteoliposomes and GUV preparation

Syt1-fl was mixed with lipids (1 mM DOPC; Avanti Research 850375) on ice in 25 mM HEPES pH 7.4, 100 mM KCl, and 0.9% CHAPS (Fisher Scientific, 50-223-7023). Total volume was adjusted to bring the detergent concentration below the critical micelle concentration. Detergent was removed by overnight dialysis at 4 °C. Proteoliposomes were then isolated by flotation of the vesicles using an Accudenz (Accurate Chemical, AN7050/BLK) step gradient.

To produce giant unilamellar vesicles (GUVs), a thin film (~15 μl) of phospholipids (DOPC, DGS-NTA, and Atto647N DOPE in the ratio 93.5:5:1.5, 1 mM total; Avanti Research 850375, 790528; Sigma 42247) was deposited onto indium tin oxide (ITO) coated glass slides. Using Nanion Vesicle Prep Pro, the slides formed a chamber sealed by o-rings, which was filled with a 200 mM sucrose and 1 mM HEPES buffer solution. The electroformation process was initiated by applying a 10 Hz AC voltage at 3 V for 2 h at 37°C, which promotes the lipid film to hydrate and self-assemble into vesicles. The resulting GUV solution was then collected, washed in an iso-osmolar buffer to remove residual sucrose and unincorporated substances, and filtered to isolate vesicles larger than 3 μm. Atto647N-labeled GUVs were incubated with α-syt7cyto-mRuby in 25 mM Tris pH 7.4, 100 mM NaCl, and 3% PEG 8000; α-syt7cyto droplets on GUV surface were imaged in a Bioinert μ-dish (Ibidi, 81150) using confocal microscopy. All consumables were brought from Nanion Technologies GmbH.

### Hydropathicity analysis

The hydropathicity scores for the juxtamembrane linkers of α-, β-, and γ-syt7 (table S1) were calculated using: https://web.expasy.org/protscale/. These scores are based on Kyte and Doolittle scoring index. A hydropathicity score of 0.25 was used as a cut-off; a high hydropathicity index indicates high hydrophobicity.

### Antibodies

#### Primary Antibodies:

**Table:** 

Antibody	Source	Identifier	Concentration
Anti-syt7 (Rabbit pAb)	SYSY	105–173; RRID: AB_887838	WB: 1:3000
Anti-syt7 (Rabbit mAb)	AbCam	ab311843	WB: 1:2000
Anti-FLAG (Rabbit mAb)	Cell Signaling Technology	D6W5B, 14793; RRID: AB_2572291	WB: 1:1000 ICC: 1:500 IP: 1:40 MINFLUX: 1:2500
Anti-Rho1D4 (Mouse mAb)	University of British Columbia	RHO 1D4	IP: 1:40
Anti-SNAP25 (Rabbit mAb)	AbCam	EPR3275; RRID: AB_10887757	WB: 1:1000
Anti-syb2 (Mouse mAb)	SYSY	104 211; RRID: AB_2619758	WB: 1:1000
Anti-syntaxin (Mouse mAb)	SYSY	110 011; RRID: AB_887844	WB: 1:1000
Anti-SV2 (Mouse mAb)	Developmental Studies Hybridoma Bank (DSHB)	SV2; RRID: AB_2315387	WB: 1:500
Anti-VGlut1 (Mousse mAb)	NeuroMab	VGlut1; RRID: AB_2187693	ICC: 1:500
Anti-Pan-Neurofascin (Mousse mAb)	NeuroMab	Pan-Neurofascin (extracellular); RRID: AB_2187693	Live imaging: 1:100
Anti-syt1 (Mouse mAb)	DSHB	MAB48 (asv 48); RRID: AB_2199314	WB: 1:1000 MINFLUX: 1:2000
Anti-Doc2a/b (Rabbit pAb)	SYSY	174 203; RRID: AB_11064600	WB: 1:250
Anti-Bassoon (Guinea pig pAb)	SYSY	141 005; RRID: AB_2924946	ICC: 1:250
Anti-Syp (Guinea pig mAb)	SYSY	101 308; RRID: AB_2924959	WB: 1:500

#### Secondary Antibodies:

**Table:** 

Antibody	Source	Identifier	Concentration
Goat anti-Rabbit IgG HRP	Bio-Rad	1706515; RRID: AB_11125142	WB: 1:10,000
Goat anti-Mouse IgG-HRP	Bio-Rad	1706516; RRID: AB_2921252	WB: 1:10,000
Goat anti-Mouse IgG2α-Alexa Fluor 647	Thermo Fisher Scientific	A21241; RRID: AB_2535810	ICC (1:500)
Goat anti-Rabbit IgG-Alexa Fluor 546	Thermo Fisher Scientific	A11035; RRID: AB_143051	ICC (1:500)
Goat anti-Guinea Pig IgG-Alexa Fluor 488	Thermo Fisher Scientific	A11073; RRID: AB_2534117	ICC (1:500)
Goat anti-Guinea Pig IgG HRP	AbCam	Ab6908; RRID: AB_955425	WB: 1:10,000
Goat anti-Mouse IgG-Flux 640	Abberior	FX 640	MINFLUX: 1:2500
Goat anti-Rabbit IgG-Flux 680	Abberior	FX 680	MINFLUX: 1:3000

## Supplementary Material

Supplement 1

## Data Availability

All materials, gels, blots, and statistical information are available in the supplemental materials. Raw data is available on Dryad, a publicly available repository. Code is available on GitHub. All other information is available in the main text or supplemental material. Dryad:http://datadryad.org/share/LINK_NOT_FOR_PUBLICATION/BDtrDQSWRIZdXvUczztX6v2f9ldyTiiNUD490ZRUhsE GitHub: https://github.com/nikunjrmehta/MINFLUX-Data-Analysis.git
