# Supplementary material for: Alternative splicing of synaptotagmin 7 regulates oligomerization and short-term synaptic plasticity
Source: bioRxiv. 2025 Oct 28:2025.10.27.684894. Preprint. [Version 1] doi: 10.1101/2025.10.27.684894 (PMC12636619; doi:10.1101/2025.10.27.684894)
Supplement: Supplement 1 [file media-1.pdf]

Supplementary Figures

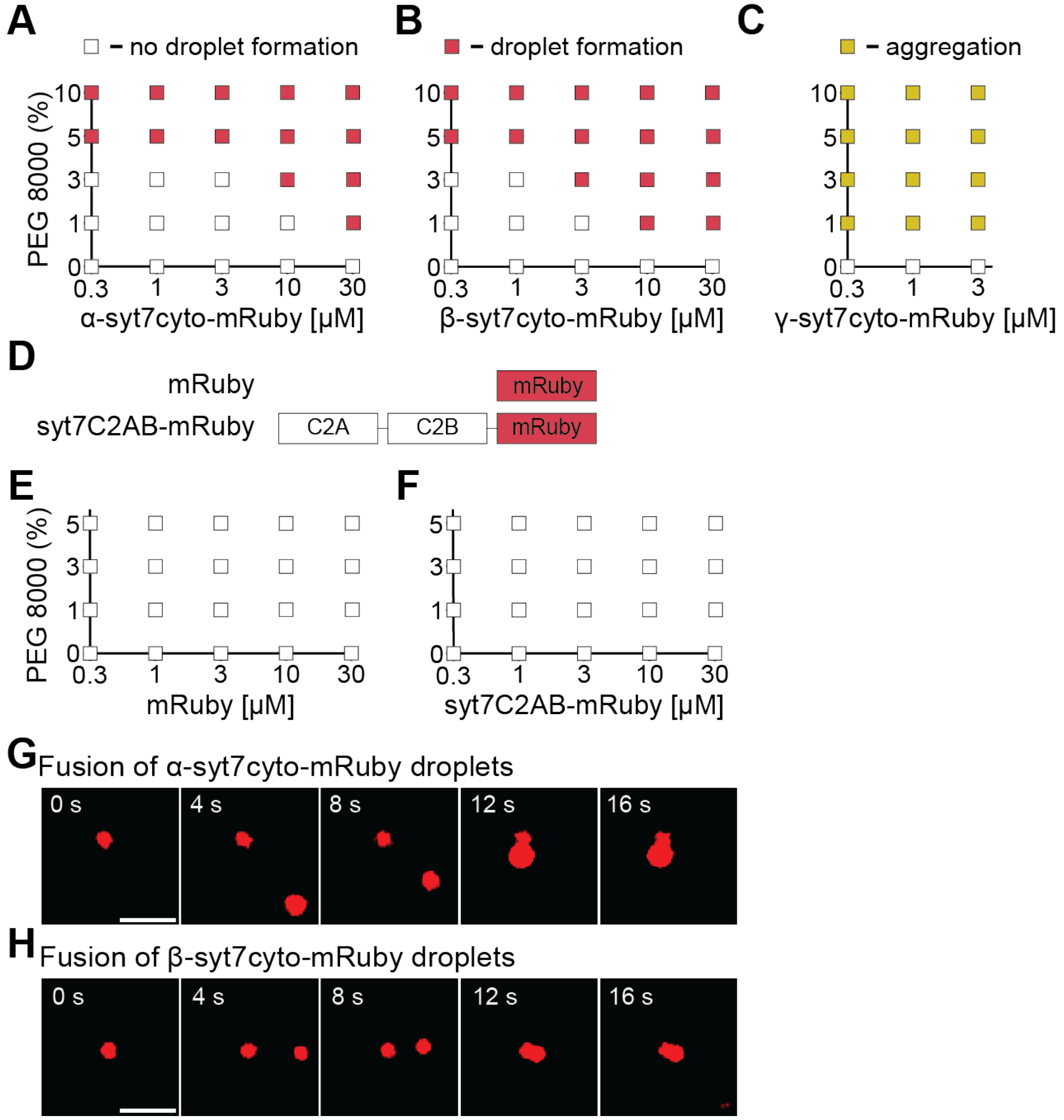

Fig. S1. Phase diagram of α, β, and γ-syt7cyto variants and fusion of α and β-syt7cyto droplets.

(**A-C**) Phase diagram of alternative splice variants of syt7 (α-, β-, and γ-syt7cyto, respectively) at indicated [protein] and [PEG 8000]. Red and yellow squares indicate the formation of droplets and aggregates, respectively, whereas empty squares indicate no effect. Note, the β variant had a slightly higher propensity to form droplets than α-syt7cyto, whereas γ-syt7cyto formed aggregates under all PEG 8000 conditions. (**D**) Control constructs: fluorescent marker, mRuby and syt7C2AB-mRuby. (**E-F**) Phase diagram of the controls in (D) at indicated [protein] and [PEG 8000], with a similar color-scheme as (A-C). No droplets or aggregates were observed for the controls. (**G,H**) Fusion of α- and β-syt7cyto droplets over time. Scale bar, 4 µm (G,H). For all experiments, N≥3 FOVs from three independent trials. The buffer used in (A-C,E,F) was 25 mM Tris-HCl (pH 7.4), 100 mM NaCl, and the indicated % PEG 8000.

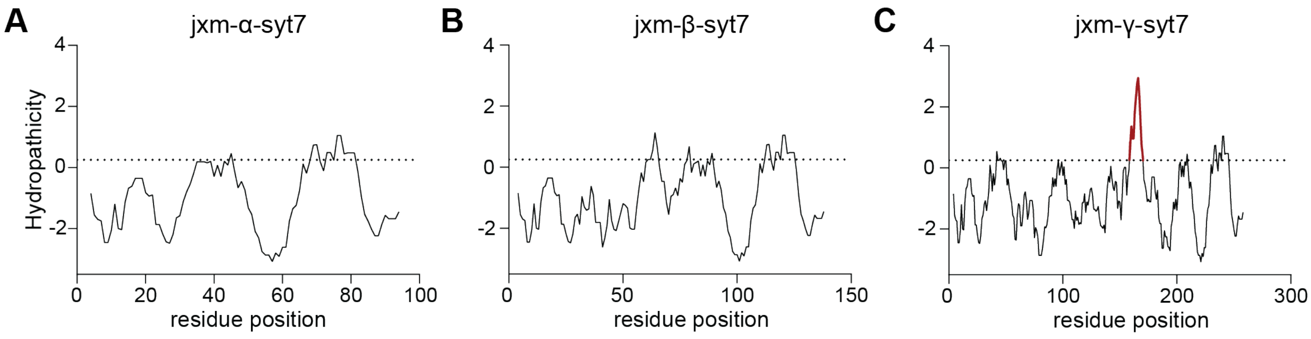

Fig. S2. Hydropathicity index of the juxtamembrane linkers of syt7 alternative splice variants.

(**A-C**) Residue-based hydropathicity score of the juxtamembrane linkers of α-, β-, and γ-syt7. Scores were calculated based on Kyte and Doolittle scoring index. Note, only γ-syt7 had a significant hydropathicity index, indicated in red, for a twelve-residue segment (159- EGRMVVLSLVLG -170). A dashed line at a score of 0.25 was used as a cut-off.

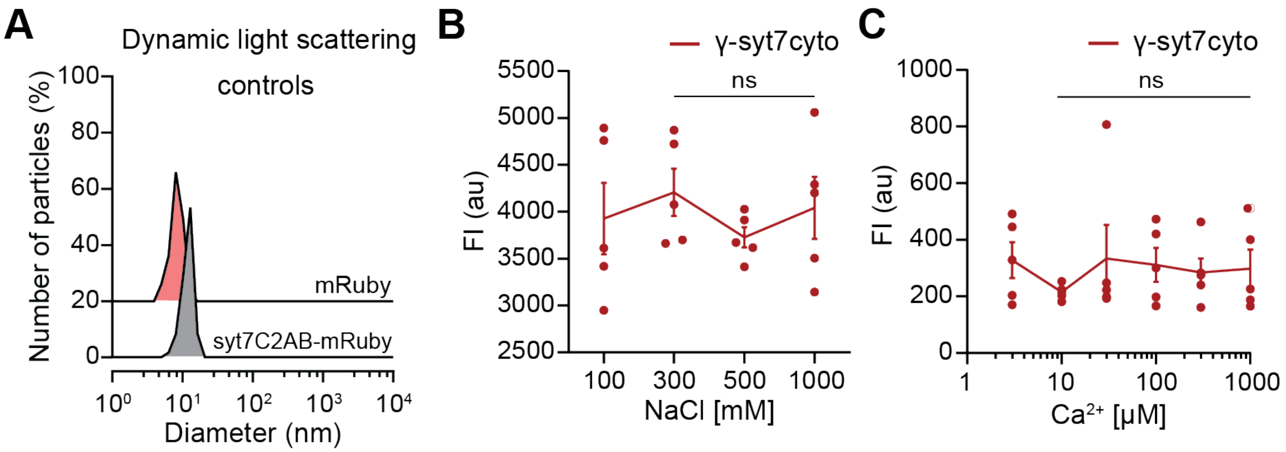

Fig. S3. DLS controls and effect of salt and Ca^{2+} on γ-syt7cyto.

(**A**) Dynamic light scattering (DLS) of the proteins illustrated in fig. S1D, showing number of particles (%) as a function of diameter. Both mRuby and syt7C2AB-mRuby had single peaks corresponding to monomers. (**B,C**) Effect of salt and Ca^{2+} on γ-syt7cyto aggregates, respectively; neither had an effect on the aggregates. Note the buffer used in (B) was 25 mM Tris-HCl (pH 7.4), 100 mM NaCl, and 3% PEG 8000; the same buffer, but lacking PEG 8000, was used in panel (C). N=3; five fields of view (FOVs) were analyzed for each condition tested; data are represented as mean ± SEM. ns indicates not significant.

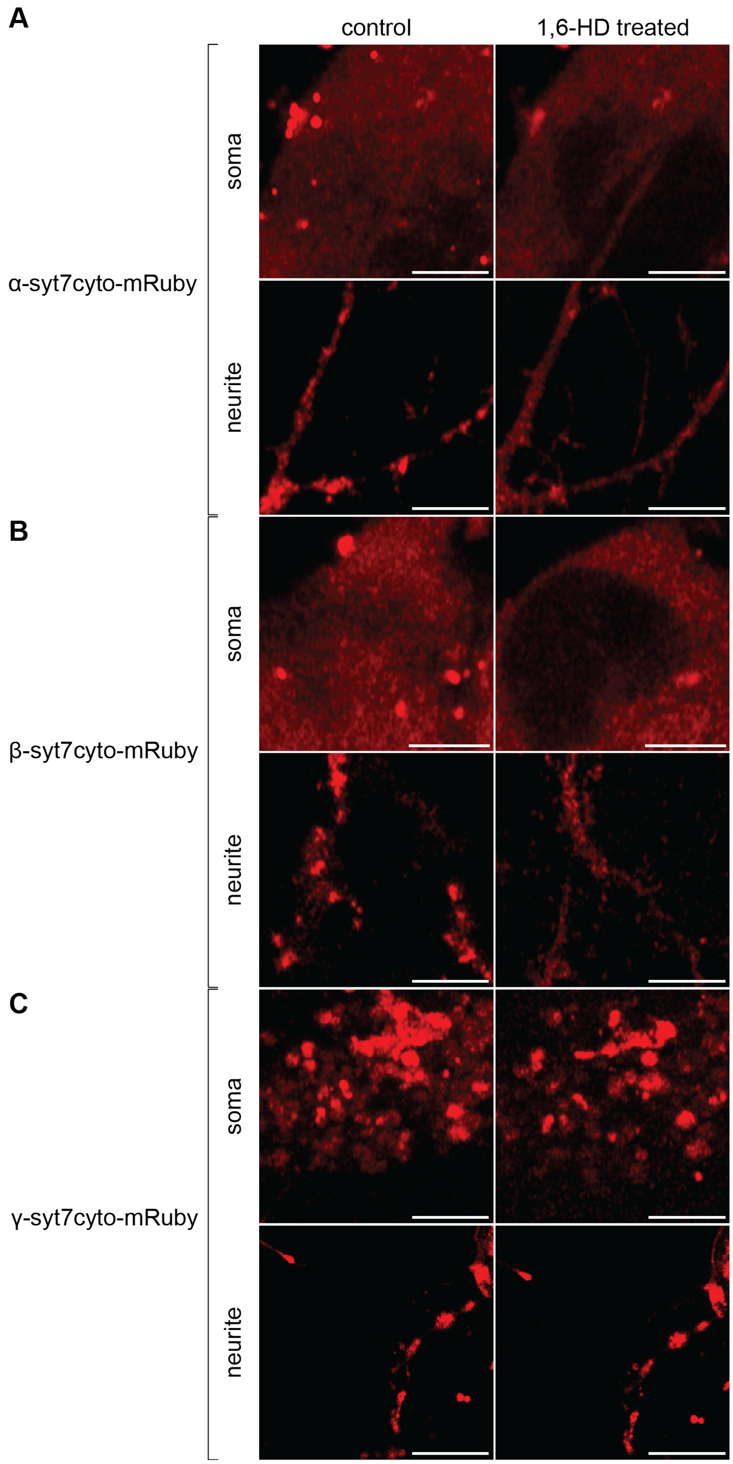

Fig. S4. α, β, and γ-syt7cyto form clusters in rat hippocampal neurons.
(**A-C**) Fluorescence images, captured on a confocal microscope, of the soma and neurites of rat hippocampal neurons transfected with α, β, or γ-syt7cyto-mRuby, respectively, under control and 10% 1,6-HD conditions. Upon 1,6-HD treatment, α- and β-syt7cyto droplets were reduced, while γ-syt7cyto aggregates were unaffected. Scale bar, 4 μm (A-C). N=3 from three independent cultures.

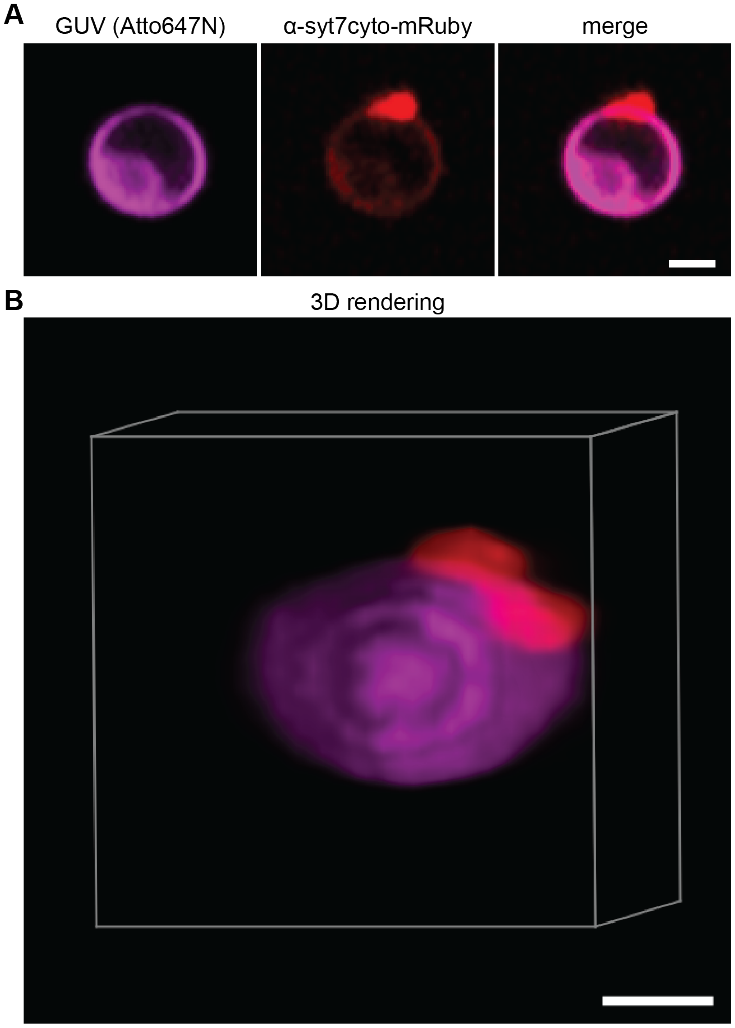

Fig. S5. α-syt7 2D liquid-liquid phase separation on the surface of a GUV.

(**A**) A Z-section plane of an Atto-647-labeled GUV (93.5% DOPC, 5% 18:1 DGS-NTA, 1.5% Atto647N DOPE; magenta) and α-syt7cyto-mRuby (red) captured on a confocal microscope. 2D LLPS of the α-syt7cyto-mRuby 'droplet' on the surface of GUV is apparent. Scale bar, 10 μm. (**B**) 3D rendering of the 2D LLPS of α-syt7cyto-mRuby 'droplet' on the surface of GUV. Scale bar, 2 μm. N=3 independent trials.

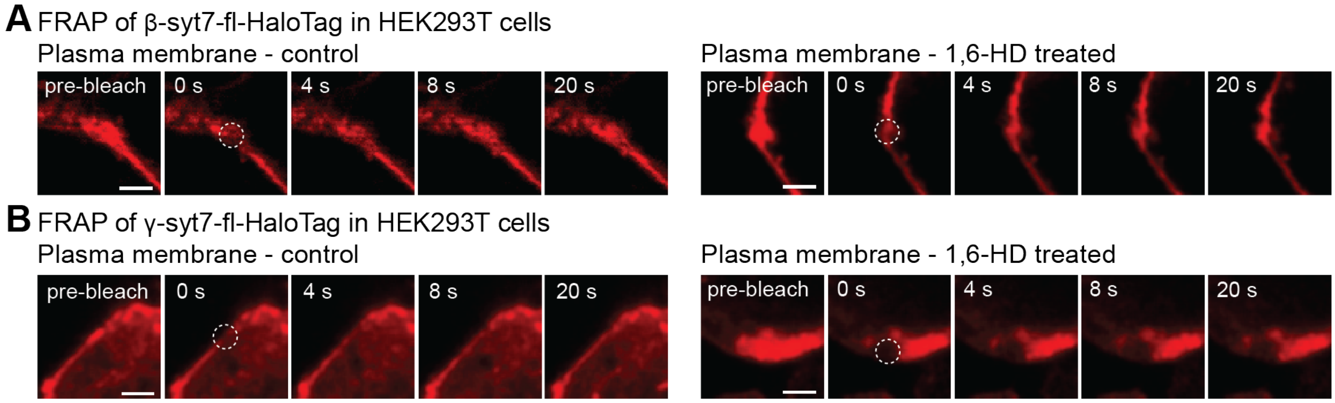

Fig. S6. FRAP of β- and γ-syt7-full length on the plasma membrane in HEK293T cells.

(**A**) Time series of fluorescence recovery after photobleaching (FRAP) at the plasma membrane of HEK293T cells expressing β-syt7-full length(fl)-HaloTag, under control and 10% 1,6-HD conditions, respectively. Scale bar, 2 µm. (**B**) same as (A), but for γ-syt7-fl-HaloTag. Note that β-syt7, but not γ-syt, recovered. Scale bar, 2 µm. N≥10-15 bleached regions across conditions from three independent trials. HaloTag-fusion proteins were labeled with JF549 fluorescent dye.

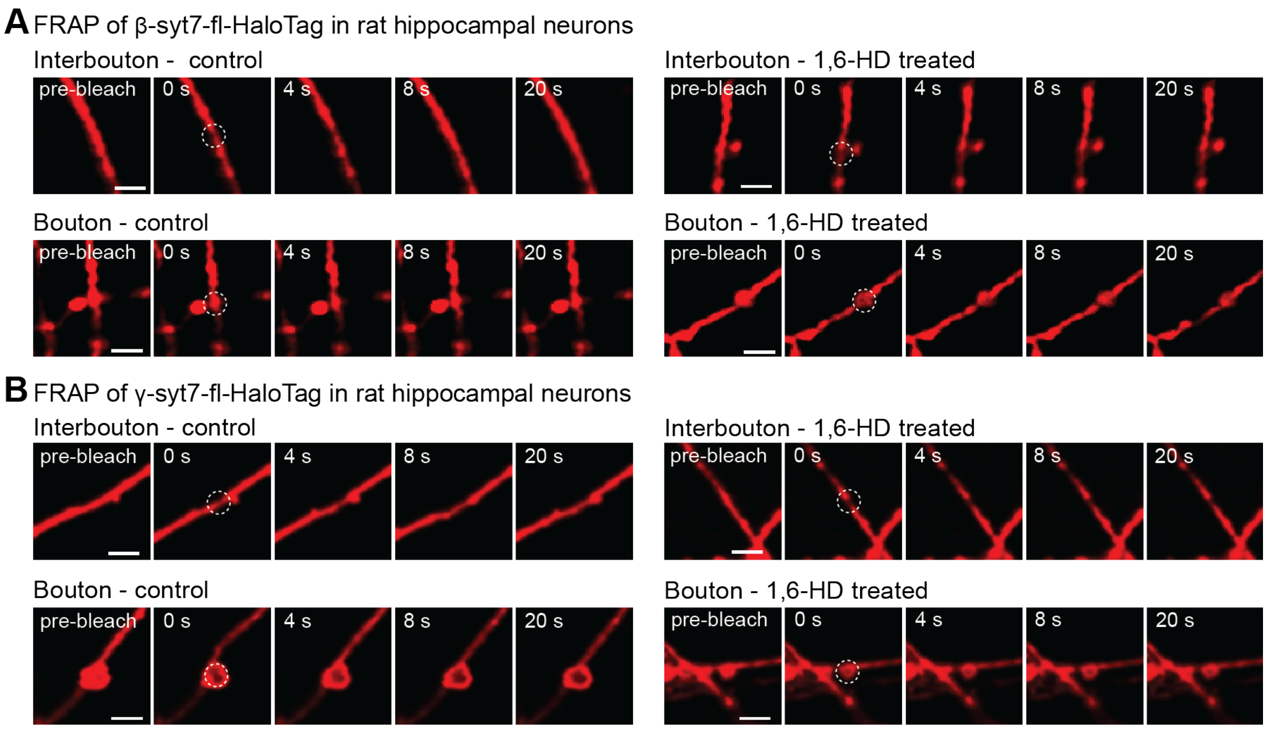

Fig. S7. FRAP of β- and γ-syt7-full length in synaptic boutons and interbouton regions of hippocampal neurons.

(**A**) Time series of FRAP at the indicated locations in rat hippocampal neurons expressing β-syt7-fl-HaloTag, under control and 10% 1,6-HD conditions. Scale bar, 2 μm. (**B**) same as (A), but for γ-syt7-fl-HaloTag. Scale bar, 2 μm. Neither isoform recovered from FRAP in boutons, while β-syt7, but not γ-syt7, recovered within the interbouton regions. Scale bar, 2 μm. N≥10-15 bleached regions across conditions from three independent trials. HaloTag-fusion proteins were labeled with JF549 fluorescent dye.

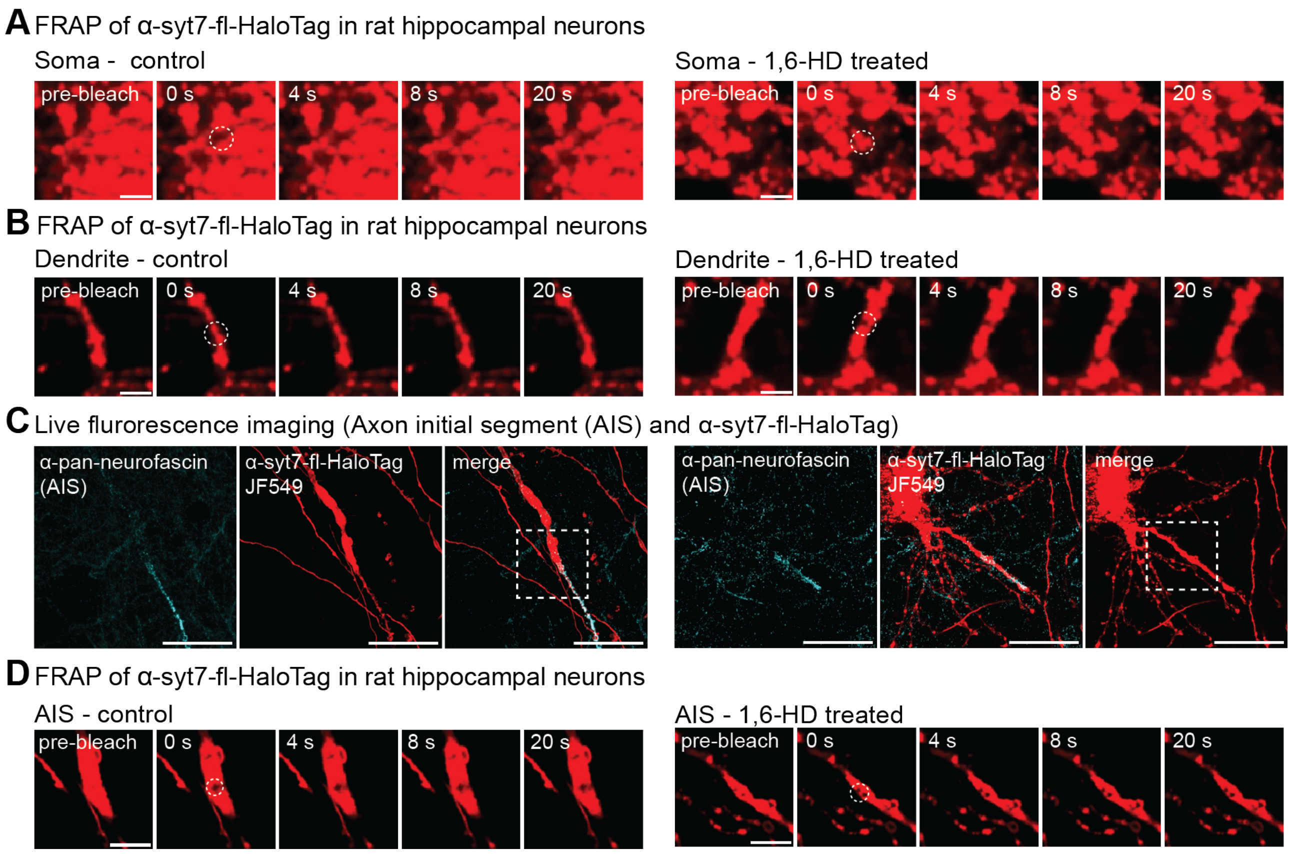

Fig. S8. FRAP of α-syt7-full length in the soma, dendrites, and AIS of hippocampal neurons.

(**A,B,D**) Time series of FRAP at the soma, dendrite, and axon initial segment (AIS), respectively, under control and 1,6-HD conditions. While syt7 is mainly an axonal plasma membrane protein in neurons, overexpression results in spill-over into the somato-dendritic compartment. Surprisingly, this mistargeted protein is relatively immobile, as the bleached area recovered less than 15% across all conditions. Scale bar, 2 μm (A,B), 5 μm (D). (**C**) Live cell imaging of rat hippocampal neurons expressing α-syt7-fl-HaloTag, visualized using JF549 fluorescent dye. AIS was marked extracellularly on live cells with anti-pan-neurofascin antibody, followed by AF647 secondary antibody labeling. Scale bar, 20 μm. N≥10-15 bleached regions across conditions from three independent trials. Box indicated the FOV used for bleaching in (D).

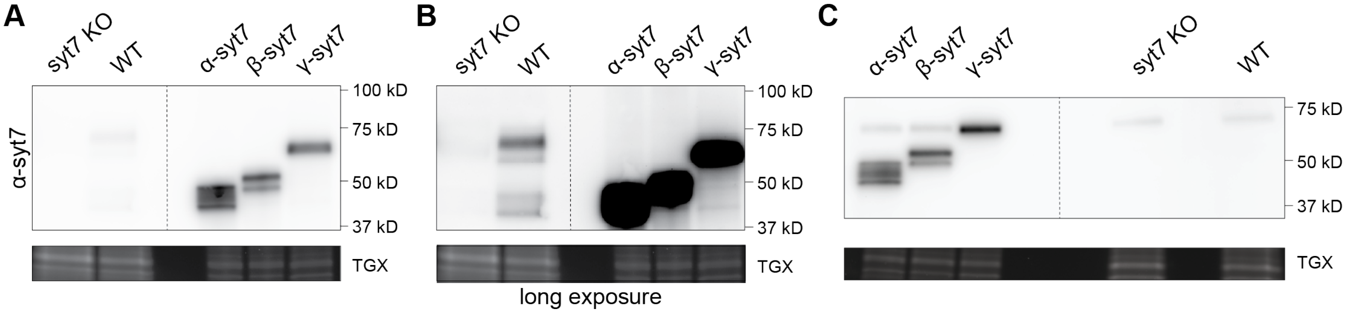

Fig. S9. Immunoblotting of syt7 splice variants.

(**A**) Representative western blot from 15 days *in vitro* (DIV) lysates from cultured mouse hippocampal neurons showing syt7 KO (CRE-treated), WT, and syt7 KOs expressing α, β, or γ-syt7, along with a loading control, imaged using stain-free method (indicated as TGX, tris-glycine extended method, BioRad). Probing with an anti-syt7 antibody (SYSY) confirmed KO of syt7, but the syt7 band was weak. (**B**) With long exposure, the same blot clearly shows KO of syt7. SYSY antibody is sensitive to the juxtamembrane linker and thus recognizes the alternative splice variants differentially. (**C**) Probing with anti-syt7 (AbCam) antibody, which specifically recognizes the C2 domains, and does not differentiate between the alternative splice variants, shows expression of the three splice variants, with equal loading in (A). Note that the AbCam antibody is not sensitive enough to detect syt7 WT levels. From densitometry analysis in (A), α-syt7 was expressed ~64 times more than WT. With equal protein loading, (B) shows β-and γ-syt7 were expressed at ~0.9 and ~0.8 expression levels of α-syt7. N≥3 blots, with three independent cultures.

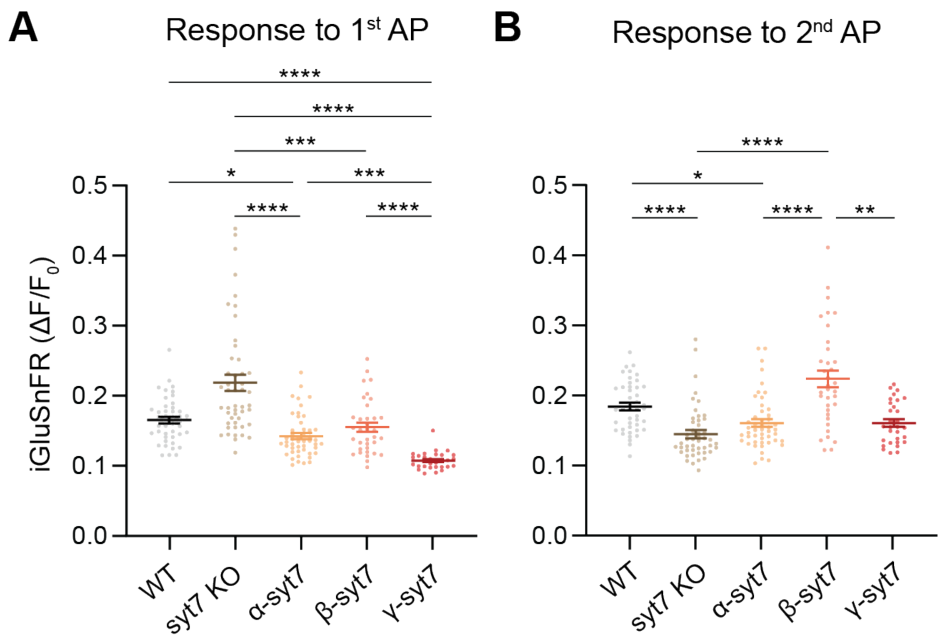

Fig. S10. iGluSnFR signal in response to the first and second action potential. (**A,B**) Quantification of peak iGluSnFR first and second responses ($\Delta F/F_0$), respectively, in the first 10 ms bin after applying two APs separated by 50 ms. As compared to the WT condition, the responses to the first stimulus were significantly lower for α- and γ-syt7. Number of FOVs analyzed: 44, 48, 48, 35, and 30 for WT, syt7 KO, α-, β-, or γ-syt7 conditions, respectively, across three or more independent culture preparations; data are represented as mean ± SEM. To determine statistical significance, one-way analysis of variance (ANOVA) with Kruskal-Wallis test with Dunn's multiple comparison correction was used in (A,B). *$P < 0.05$; **$P < 0.01$; ***$P < 0.001$; ****$P < 0.0001$. Full statistics are provided in Data S2.

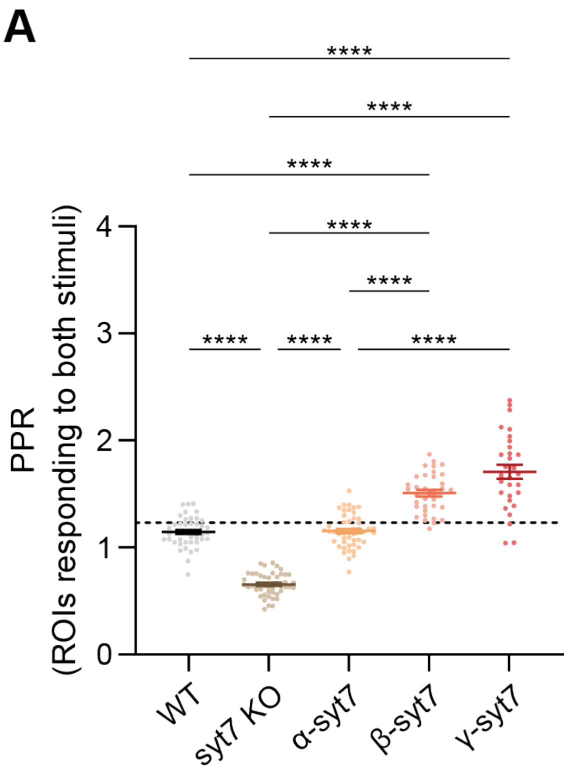

Fig. S11. iGluSnFR PPR analysis for boutons that responded to both APs.

(**A**) Quantification of paired-pulse ratio (PPR) of peak iGluSnFR responses ($\Delta F/F_0$) from ROIs responding to both first and second stimuli at 20 Hz. Dashed line indicates WT PPR from Fig. 3B. Note: a similar trend is observed as Fig. 3B. PPRs calculated from ROIs responding to both stimuli contribute 94, 95, 92, 90, and 71% to the combined PPR of the five conditions tested. Number of FOVs analyzed: 44, 48, 48, 35, and 30 for WT, syt7 KO, α-, β-, or γ-syt7 conditions, respectively, across three or more independent culture preparations; data are represented as mean ± SEM. To determine statistical significance, one-way analysis of variance (ANOVA) with Kruskal-Wallis test with Dunn's multiple comparison correction was used in (A). **P* < 0.05; ***P* < 0.01; ****P* < 0.001; *****P* < 0.0001. Full statistics are provided in Data S2.

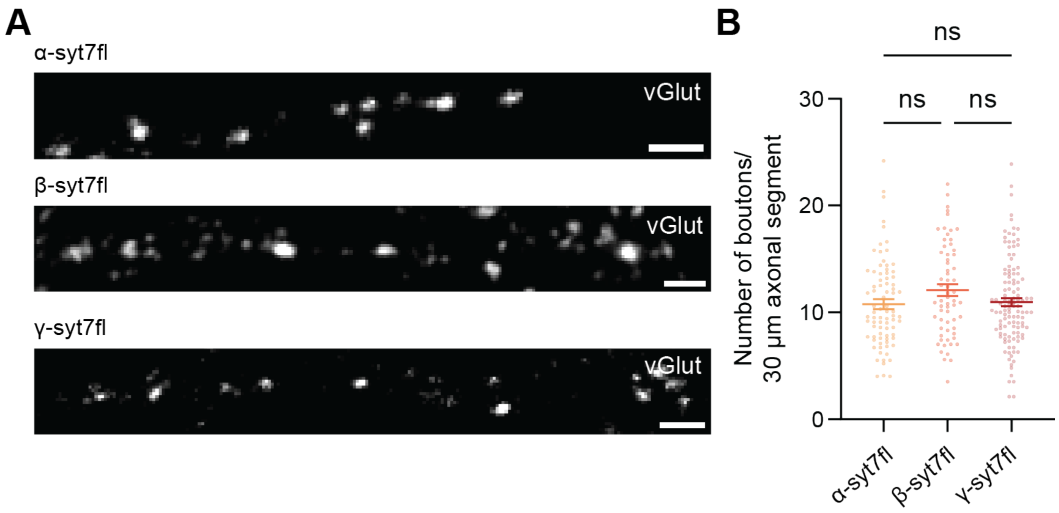

Fig. S12. A similar number of vGlut-positive synapses in syt7 KO neurons expressing each of the three splice variants of syt7.

(**A,B**) Representative fluorescence images and quantification of the number of total vGlut-positive synapses in syt7 KO hippocampal neurons expressing α, β, or γ-syt7. Neurons were stained with a vGlut antibody, and the number of puncta was quantified from multiple 30 µm axonal segments across conditions. Scale bar, 2 µm. N=10 FOVs for each condition from two culture preparations. To determine statistical significance, one-way analysis of variance (ANOVA) with Kruskal-Wallis test with Dunn's multiple comparison correction was used in (B). ns, not significant; **P* < 0.05; ***P* < 0.01; ****P* < 0.001; *****P* < 0.0001. Full statistics are provided in Data S2.

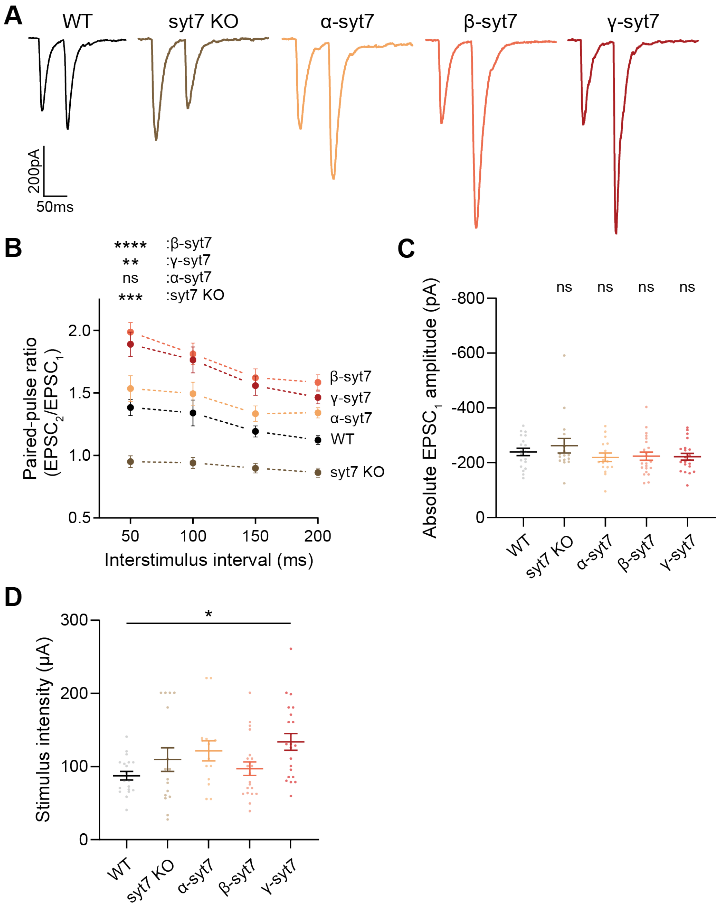

Fig. S13. Whole-cell voltage clamp recordings demonstrate that alternative splicing of syt7 regulates the degree of paired-pulse facilitation

(**A**) Representative evoked excitatory postsynaptic currents (EPSCs) traces for WT, syt7 KO, and three splice variants expressed in a syt7 KO background after paired APs at 50 ms interstimulus intervals (20 Hz). (**B**) Quantification of paired-pulse ratios of peak EPSC amplitudes ($EPSC_2/EPSC_1$) for all five conditions tested as a function of interstimulus interval (range of 50-200 ms or 5-20 Hz). (**C**) Quantification of the absolute amplitude of EPSC1 for all five conditions. Note that the $EPSC_1$ amplitudes are similar across all conditions. (**D**) Quantification of stimulus intensity set to elicit similar $EPSC_1$ amplitudes in (C) for all five conditions. Only the γ-syt7 condition was significantly higher compared to the WT condition. Number of cells: 16, 16, 16, 22, and 19 for WT, syt7 KO, α-, β-, and γ-syt7 conditions, respectively, across four independent culture preparations; data are represented as mean ± SEM. To determine statistical significance, two-way analysis of variance (ANOVA) with mixed-effects model with Geisser-Greenhouse correction using Sidaks multiple comaprison test in (B; comparison at 50 ms interstimulus shown for simplicity), one-way ANOVA with Kruskal-Wallis test with Dunn's multiple comparison test in (C), and ordinary one-way ANOVA with Tukey's multiple comparisons test with a single pooled variance in (D). ns, not significant; **P* < 0.05; ***P* < 0.01; ****P* < 0.001; *****P* < 0.0001 as compared to WT condition. Full statistics are provided in Data S2.

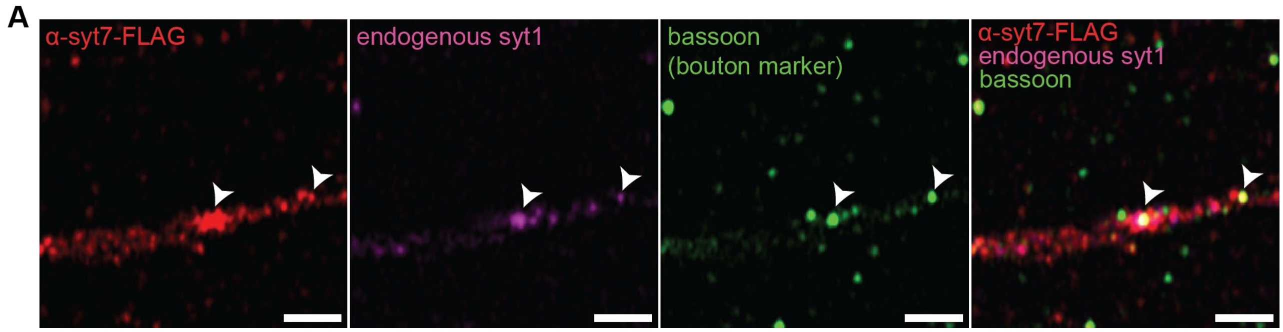

Fig. S14. α-syt7, syt1, and bassoon colocalize at synapses using confocal fluorescence microscopy.

(**A**) Representative confocal images of α-syt7FLAG, endogenous syt1, and endogenous bassoon (bouton marker) in red, magenta, and green, respectively, marked using primary antibodies (anti-FLAG (M2 Rabbit), anti-syt1 (mAb48), and anti-bassoon (SYSY); see Antibodies section for detailed information); a merged image is shown in the right, indicated some degree of colocalization at synapses, as indicated by the arrows. Scale bar, 2 μm.

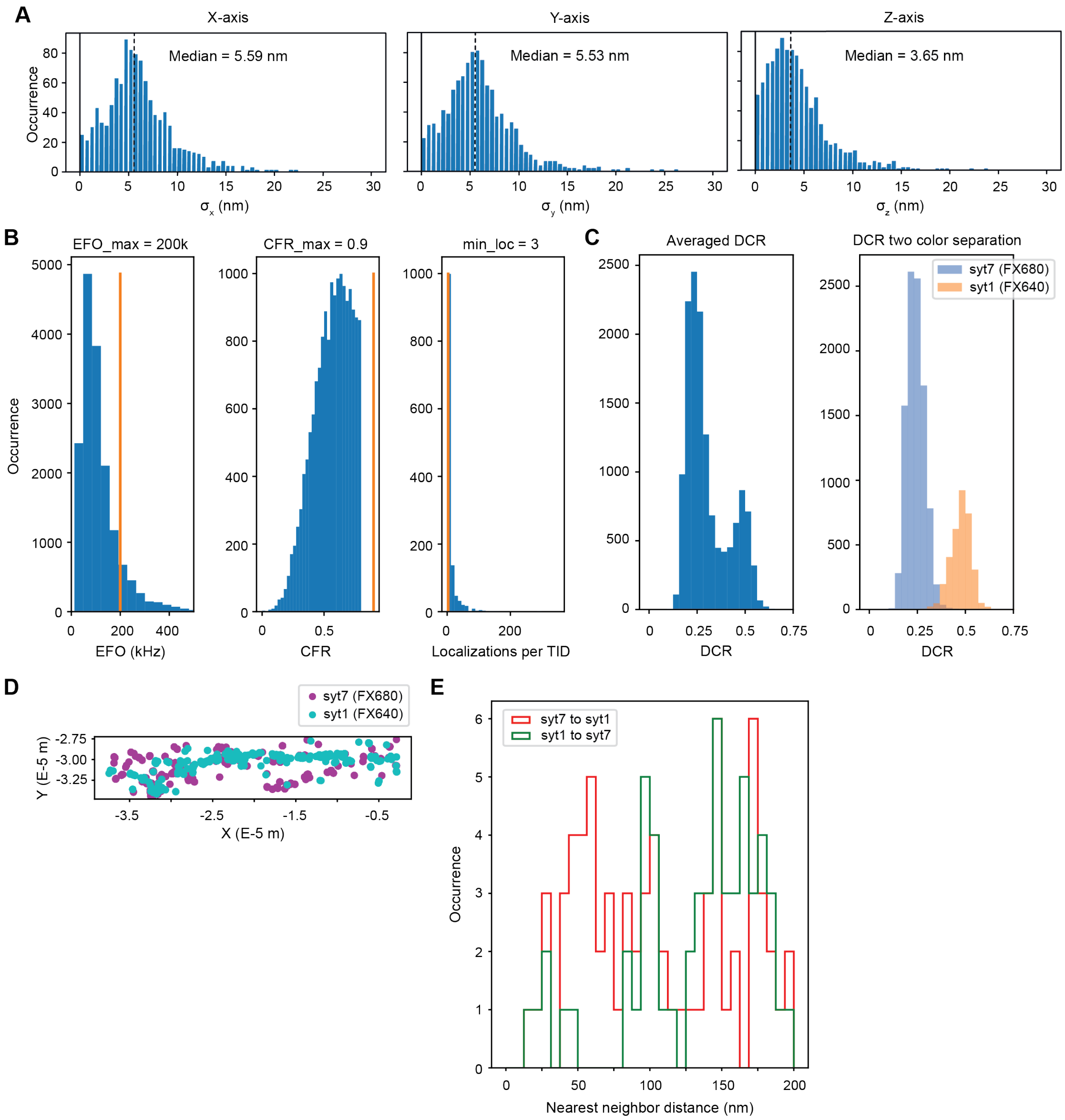

Fig. S15. Resolution and filtering of MINFLUX data.

(**A**) Representative histogram of standard deviations (σ) of localization precision along X, Y, and Z axes. $\sigma_x = 5.39 \pm 0.26$, $\sigma_y = 5.14 \pm 0.26$, and $\sigma_z = 3.01 \pm 0.22$. (**B**) Representative histograms from a 2-color 3D MINFLUX image showing effective frequency at offset (EFO), center frequency ratio (CFR), localization per TIDs (Trace IDs) set as EFO_max = 200k Hz, CFR_max = 0.9, and localization when TID > 3. After filtering the data, (**C**) the detection channel ratio (DCR) was calculated by spectral unmixing of the average DCR plot. The two peaks were fitted with Gaussian functions, indicative of the FX680 (syt7) and FX640 (syt1) dyes, respectively. (**D**) DCR-based color assignment was used to separate FX640 and FX680 in cyan and magenta, and (**E**) nearest neighbor distances were plotted for FX640 to FX680 (indicated as syt7 to syt1; red) and vice-versa (indicated as syt1 to syt7; green), as described in methods. N=15 images from 5 independent culture preparations. Data are represented as mean ± SEM.

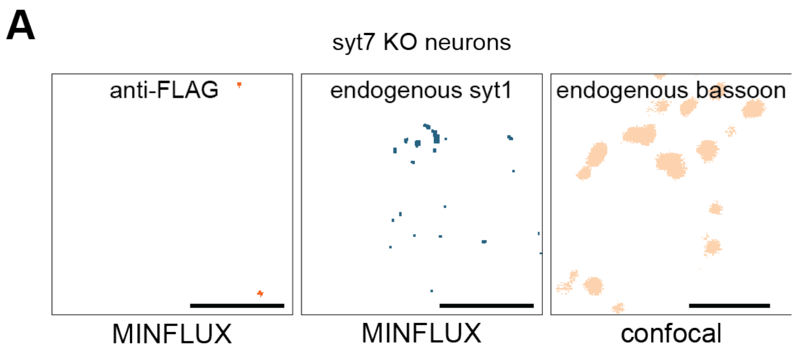

Fig. S16. MINFLUX imaging controls: non-transduced syt7 KO neurons

(**A**) Representative images of maximum Z-projection of syt7 (stained with an anti-FLAG antibody in orange, MINFLUX), syt1 (stained with mAb48 in blue, MINFLUX), and bassoon (stained with SYSY 141005 in tan, confocal) from a FOV of non-transduced syt7 KO mouse hippocampal neurons. In the absence of transduced FLAG-tagged syt7, little signal was observed using the anti-FLAG antibody. Endogenous syt1 and bassoon expression were normal in the syt7 KO neurons. Scale bar, 2 µm.

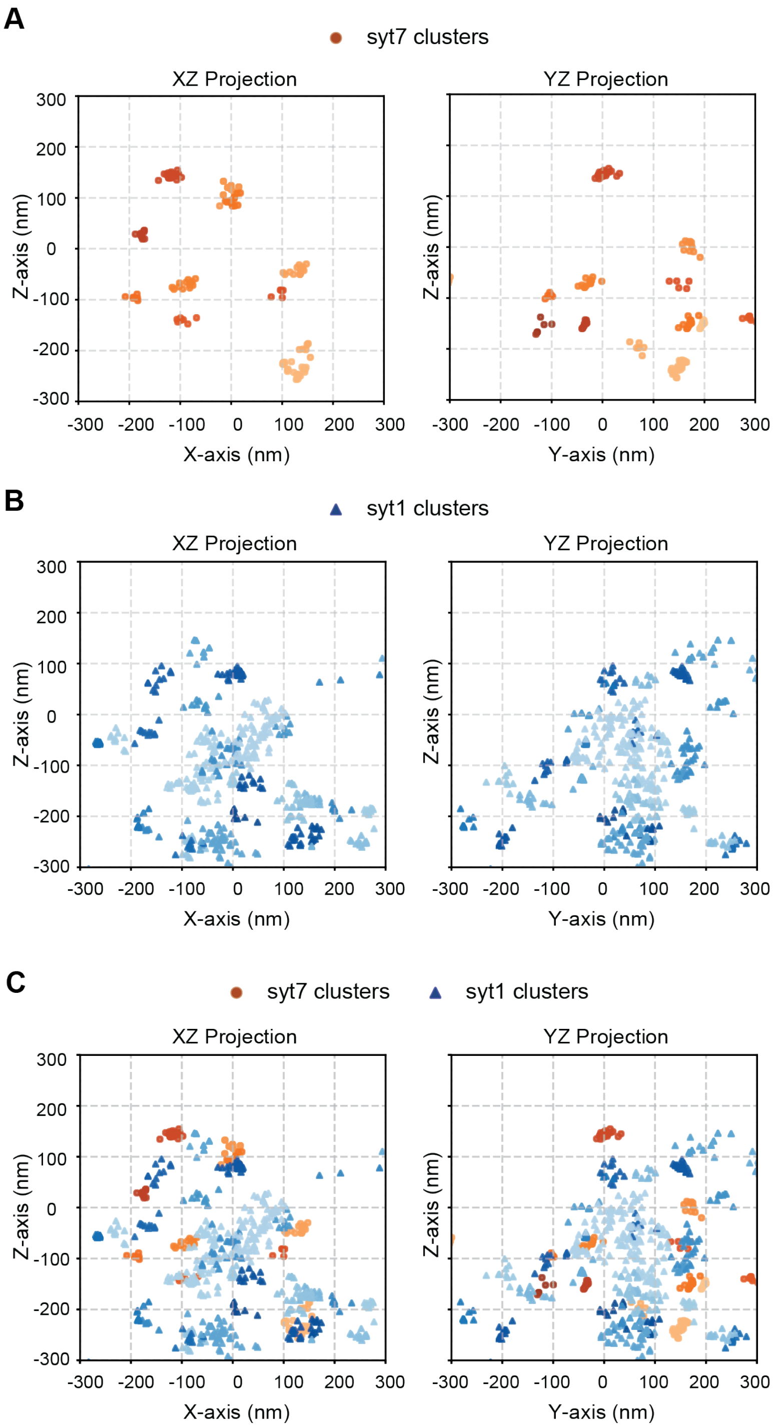

Fig. S17. 2D projections from 3D scatter plots of syt7 and syt1 clusters along the XZ and YZ axes.

(**A-C**) 2D scatter plot generated from the 3D graph shown in Fig. 5G, of syt7 and syt1 clusters, illustrating the projections along the XZ and YZ axes. The center (0,0) indicates the centroid of the bassoon obtained from confocal imaging. Syt7 and syt1 clusters are represented in orange circles and blue triangles, respectively. Note, the clusters are grouped using the same shade of color.

A

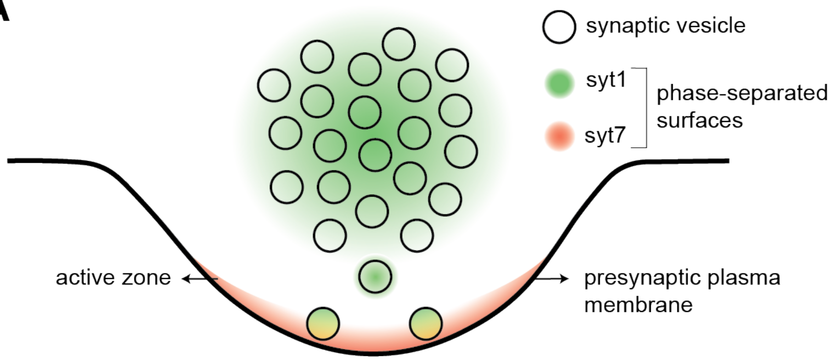

Fig. S18. Model describing how syt7-syt1 interactions might contribute to SV docking

(A) Schematic of SV docking at the active zone of the presynaptic plasma membrane at a bouton. SVs are denoted by circles; syt1 phase-separated surface in green, and syt7 phase-separated surface in red. Syt7-syt1 LLPS ‘surfaces’ adhere to each other, contributing to another protein-protein interaction in the docking pathway. Since syt7 KO neurons do not show defects in docking at steady state (Vevea et al., 2021; Wu et al., 2024), we propose that the calcium-dependent increase in the avidity of this interaction, likely mediated by the C2-domains (Fukuda et al., 2002), contributes to docking reactions during activity. In this model, the interaction of the syt7 and syt1 jxm regions might serve to poise the C2-domains for rapid, efficient interactions during ongoing activity.

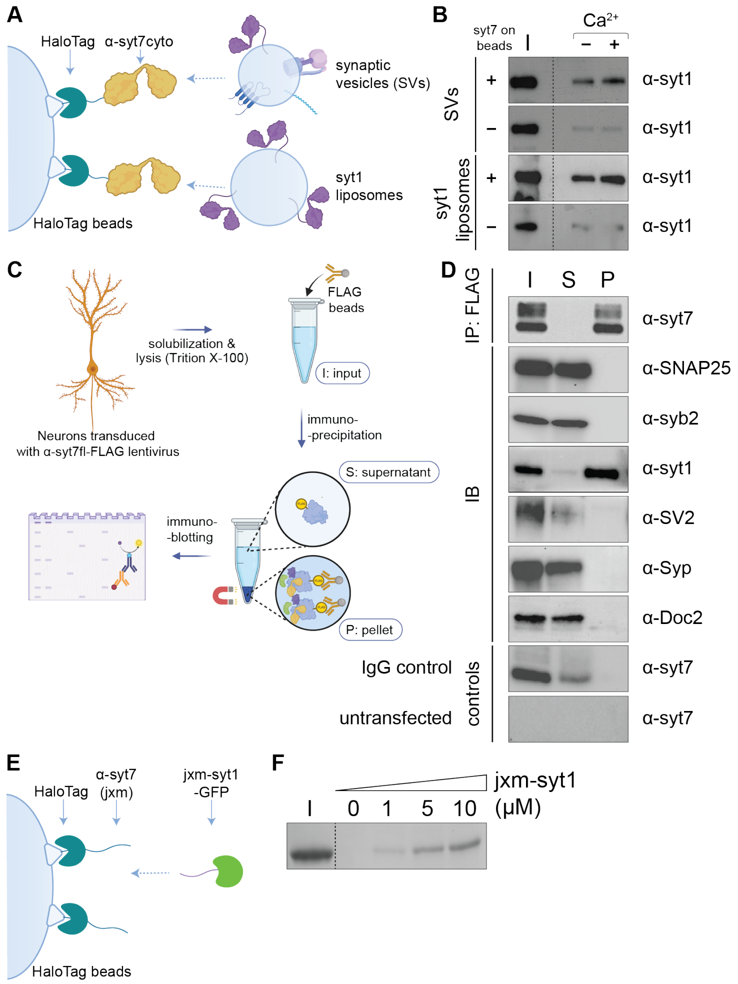

Fig. S19. Direct interaction between the jxm linkers of α-syt7 and syt1, and α-syt7 directly binds purified synaptic vesicles (SV).

(**A**) Schematic of the immunoprecipitation (IP) procedure showing neurons transduced with α-syt7-fl-FLAG lentivirus. The lysates (input) were incubated with anti-FLAG-conjugated magnetic Dynabeads to selectively pull-down α-syt7FLAG and its binding partners (pellet). IP'ed α-syt7FLAG, and potential binding partners were detected via Western blot analysis. The IP supernatant was included to assay for depletion of any bound species. (**B**) Blots showing successful pull-down of α-syt7FLAG. Only syt1 co-IP'ed; SNAP-25, syb2, SV2, Syp, and Doc2 failed to bind, suggesting a specific interaction between α-syt7FLAG and syt1. Two controls were used: IgG-conjugated Dynabeads and untransfected neurons; α-syt7-FLAG did not IP in either case. (**C,D**) Schematic and representative immunoblots from a HaloTag pull-down assay showing that α-syt7cyto directly binds to both purified SVs from mouse brain, as well as syt1-fl reconstituted into liposomes, in a weakly Ca^{2+}-dependent manner (SVs: 1.34 ± 0.23, syt1 liposomes: 1.46 ± 0.15). Empty beads showed minimal binding. (**E,F**) Schematic and representative gels from a HaloTag pull-down assay showing that direct binding occurs between the jxm linkers of syt7 and syt1 in a concentration-dependent manner. N=3 with three independent repeats for IP, and HaloTag pull-down assays, data are represented as mean ± SEM.

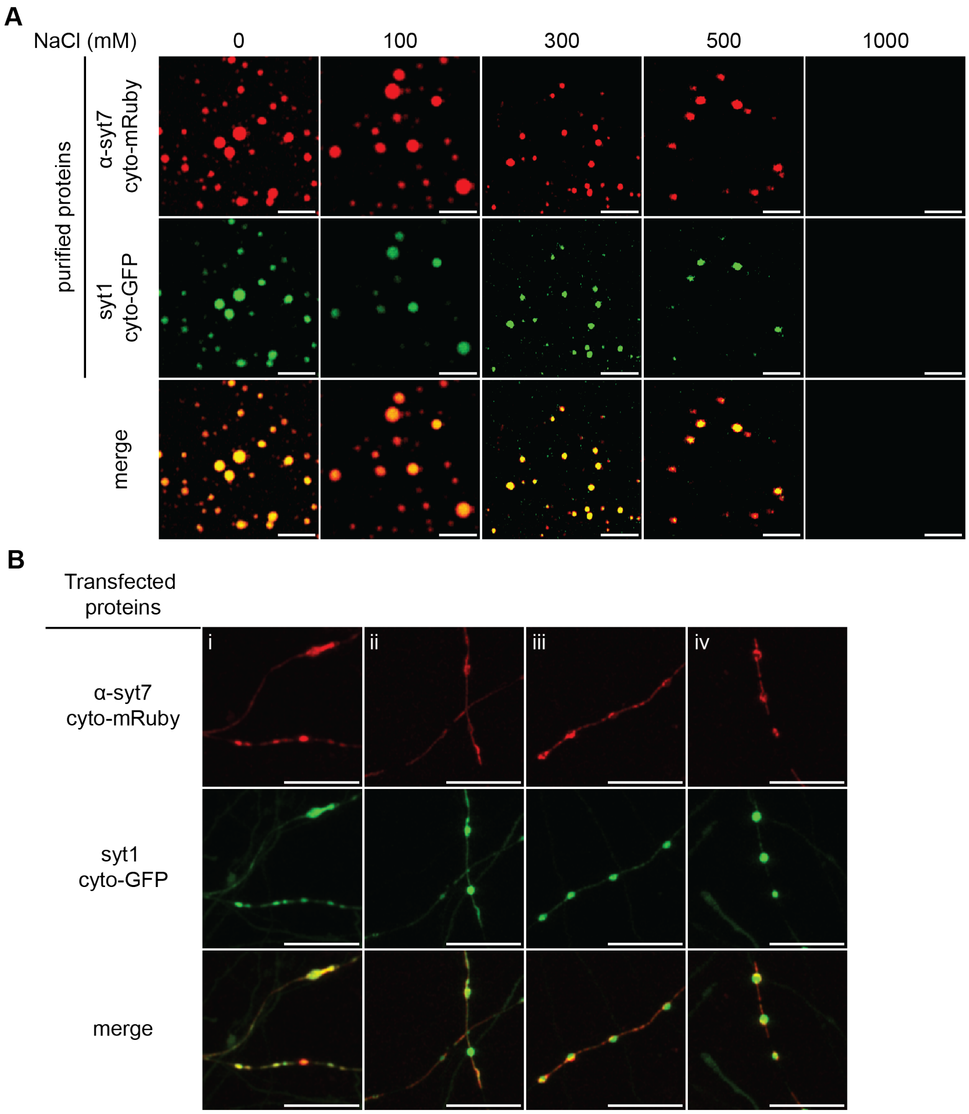

Fig. S20. α-syt7cyto and syt1cyto droplets coalesce *in vitro* and in hippocampal neurons.

(**A**) Representative confocal image showing α-syt7cyto and syt1cyto droplets coalescing in the 0 mM NaCl condition. With increasing ionic strength, the coalescent α-syt7cyto and syt1cyto droplets dissolve, suggesting ionic interactions within droplets. Scale bar, 5 μm. (**B**) Representative images (i-iv) of rat hippocampal neurons transfected with α-syt7cyto-mRuby and syt1cyto-GFP illustrating that these droplets coalesce in neurites. Scale bar, 15 μm. N=3 with three independent repeats. The buffer used in (A) was 25 mM Tris-HCl (pH 7.4), indicated NaCl, and 3% PEG 8000.

Table S1.

Sequences of alternative splice isoforms of syt7

Syt7 alternative splice isoforms	Juxtamembrane linker protein sequence	# of residues
α-syt7	**C**HW**C**QRKLGKRYKNSLETVGTPDSGRGRGEKKAIKL PAGGKAVNTAPVPGQTPHDESDRRTETRSSVSDLVNS LTSEMLMLSPGSEEDEAHEGCSRENL	99
β-syt7	**C**HW**C**QRKLGKRYKNSLETVGTPDSGRGRGEKKAIND LDRDFWNNNESTVQQKWSSYPPKEFILNISPYAPYGD PRLSLKLPAGGKAVNTAPVPGQTPHDESDRRTETRSS VSDLVNSLTSEMLMLSPGSEEDEAHEGCSRENL	143
γ-syt7	**C**HW**C**QRKLGKRYKNSLETVGTPDSGRGRGEKKAINF EDSTLSTATTLESIPSSAGEPKCQRPRTLMRQQSLQQ PLSQNQRGRQPSQPTTSQSLGQLQAHAASAPGSNP RAYGRGQARQGTSAGSKYRAAGGRSRSNPGSWDH VVGQIRNRGLDMKSFLEGRMVVLSLVLGLSEQDDFAN IPDLQNPGTQQNQNAQGDKRLPAGGKAVNTAPVPGQ TPHDESDRRTETRSSVSDLVNSLTSEMLMLSPGSEED EAHEGCSRENL	263

Colored sequences of β- and γ-syt7 juxtamembrane linkers indicate changes in amino acid composition with respect to α-syt7. C, in bold, indicates cysteine residues which undergo palmitoylation.

Table S2.

Kinetics of FRAP recovery of each of the three alternative splice variants of syt7 at the plasma membrane in HEK293T cells

	Plasma membrane	
	Control	1,6-HD treated
α-syt7	5.43 (3.98, 6.88)	1.12 (-0.136, 2.38)
β-syt7	11.6 (6.37, 16.8)	5.48 (2.20, 8.76)
γ-syt7	2.75 (0.479, 5.01)	5.06 (0.630, 9.49)

Calculated $t_{1/2}$ (s) values from fitting FRAP recovery curves with a hyperbolic function for each of the three alternative splice variants of syt7 in HEK293T cells, under control and 1,6-HD treated conditions. Data are represented as mean and 95% confidence interval (CI).

Table S3.

Kinetics of FRAP recovery of each of the three alternative splice variants of syt7 at interbouton and bouton in rat hippocampal neurons

	Interbouton	
	Control	1,6-HD treated
α-syt7	15.3 (12.2, 18.4)	8.63 (1.70, 15.6)

β-syt7	21.8 (15.0, 28.6)	5.98 (0.497, 11.5)
γ-syt7	8.3 (1.01, 15.6)	13.6 (-1.07, 28.3)

	Bouton	
	Control	1,6-HD treated
α-syt7	6.88 (0.40, 13.4)	10.1 (2.97, 17.1)
β-syt7	4.42 (0.0121, 8.84)	4.37 (0.0165, 8.72)
γ-syt7	115 (40.9, 190)	44.2 (14.8, 73.6)

Calculated $t_{1/2}$ (s) values from fitting FRAP recovery curves with a hyperbolic function for each of the three alternative splice variants of syt7 at interbouton and bouton in rat hippocampal neurons, under control and 1,6-HD-treated conditions. Data are represented as mean and 95% confidence interval (CI).

Table S4.

Mean and amplitude for nearest neighbor distance calculations

Nearest neighbor distance (NND)	Gaussian peak	Mean ± SEM (nm)	Amplitude ± SEM
syt7 to syt1	peak 1	22.8 ± 0.42	82.5 ± 4.22
	peak 2	149 ± 3.13	29.8 ± 1.89
syt1 to syt7	peak 1	22.4 ± 1.39	33.4 ± 4.02
	peak 2	160 ± 2.83	29.6 ± 2.51

Mean and amplitude of two peaks fitted with Gaussian functions for syt7 to syt1 and syt1 to syt7 nearest neighbor distance (NND) frequency distribution graphs in Fig. 5E,F, respectively. Data are represented as mean ± SEM.

Data S1. (separate file)

Raw data and statistical analysis information for Figs. 1-5.

Data S2. (separate file)

Raw data and statistical analysis information for figs. S1-20.

Supplemental References:

Fukuda, M., Katayama, E., Mikoshiba, K., 2002. The Calcium-binding Loops of the Tandem C2 Domains of Synaptotagmin VII Cooperatively Mediate Calcium-dependent Oligomerization. Journal of Biological Chemistry 277, 29315–29320. https://doi.org/10.1074/jbc.M201697200

Vevea, J.D., Kusick, G.F., Courtney, K.C., Chen, E., Watanabe, S., Chapman, E.R., 2021. Synaptotagmin 7 is targeted to the axonal plasma membrane through γ-secretase

processing to promote synaptic vesicle docking in mouse hippocampal neurons. eLife 10, e67261. https://doi.org/10.7554/eLife.67261

Wu, Z., Kusick, G.F., Berns, M.M., Raychaudhuri, S., Itoh, K., Walter, A.M., Chapman, E.R., Watanabe, S., 2024. Synaptotagmin 7 docks synaptic vesicles to support facilitation and Doc2α-triggered asynchronous release. eLife 12, RP90632. https://doi.org/10.7554/eLife.90632
